# An Optimization Approach Coupling Preprocessing with Model Regression for Enhanced Chemometrics

**DOI:** 10.1021/acs.iecr.2c04583

**Published:** 2023-04-05

**Authors:** Chrysoula D. Kappatou, James Odgers, Salvador García-Muñoz, Ruth Misener

**Affiliations:** † Computational Optimisation Group, Department of Computing, Imperial College London, London SW7 2AZ, United Kingdom; ‡ Synthetic Molecule Design and Development, Lilly Research Laboratories, Eli Lilly & Company, Indianapolis, Indiana 46285, United States

## Abstract

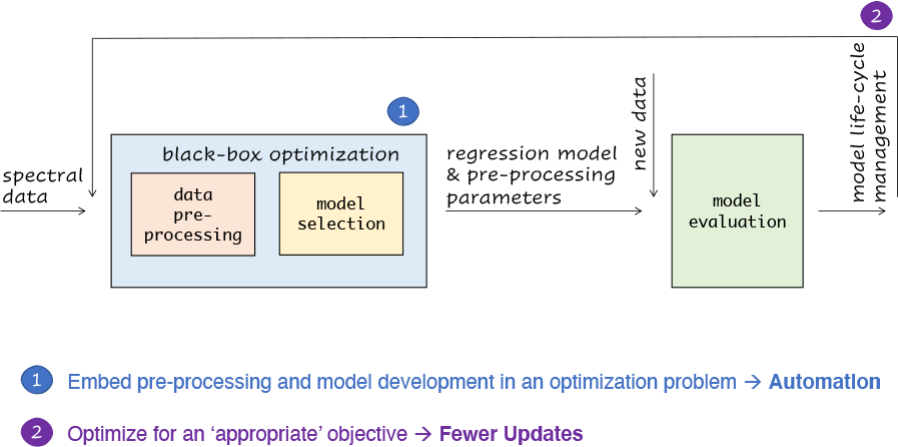

Chemometric methods are broadly used in the chemical and biochemical sectors. Typically, derivation of a regression model follows data preprocessing in a sequential manner. Yet, preprocessing can significantly influence the regression model and eventually its predictive ability. In this work, we investigate the coupling of preprocessing and model parameter estimation by incorporating them simultaneously in an optimization step. Common model selection techniques rely almost exclusively on the performance of some accuracy metric, yet having a quantitative metric for model robustness can prolong model up-time. Our approach is applied to optimize for model accuracy and robustness. This requires the introduction of a novel mathematical definition for robustness. We test our method in a simulated set up and with industrial case studies from multivariate calibration. The results highlight the importance of both accuracy and robustness properties and illustrate the potential of the proposed optimization approach toward automating the generation of efficient chemometric models.

## 1 Introduction

Chemometric data often exhibit a latent structure, meaning that the observed data can be adequately described by some nondirectly observed variables with underlying system structure. These variables are called latent variables and are useful for dimensionality reduction and interpretation. Typical examples include process analysis, monitoring and control in various chemical engineering applications, exploiting connections between chemical structure and biological activity in chemicals, and multivariate calibration.^1,2^ In multivariate calibration, signals at various wavelengths, i.e., the multivariate spectral data (regressor), are used to derive concentrations of several analytes (response) in several samples.^3^ One of the most common regression methods used in multivariate calibration is partial least squares (PLS).^4−6^

PLS regression finds a set of latent variables through projection of the regressor onto new orthogonal subspaces by maximizing the covariance between the regressor and the response latent spaces. PLS assumes an underlying linear latent structure of the data, i.e., there exists a linear combination of inputs that can effectively describe the data through their latent space.^7^ This method is most useful when the latent spaces for the regressor and the response have substantial overlap.^3^ A general description and characteristic properties of PLS regression can be found (among others) in work by Geladi and Kowalski^8^ and Wold et al.^9^

A critical step before applying PLS regression to multivariate calibration is preprocessing of the available spectral data. Data preprocessing attempts to eliminate unwanted sources of variability from the data,^10−12^ e.g., reducing measurement noise and signal interferences. A typical preprocessing strategy may include multiple stages, such as baseline and scatter correction, data smoothing, and scaling.^11,13^ There are a number of decisions associated with the preprocessing step, i.e., selecting the preprocessing stages and their order. Attempts to identify the parameters for appropriate PLS modeling lie in the general scope of hyperparameter tuning/optimization of machine learning models. Common approaches in this field include grid search, random search, and Bayesian optimization techniques such as Gaussian processes, random forests, and tree Parzen estimators.^14−17^ In general, proper selection of hyperparameters has a profound impact on model performance.^16^ In work by Bhosekar and Ierapetritou^18^ and Thebelt et al.,^19^ connections to chemical and process systems engineering are established.

In the PLS literature, the choice of appropriate preprocessing is in general case-study specific^12,13^ and is typically done on a trial-and-error basis by visual inspection or by evaluation of some quality metric on the preprocessed data.^13^ Exploring alternative approaches to make this process more rigorous is of high importance, as data preprocessing can play a crucial role on the interpretability, explainability, and eventually the predictive ability of a chemometric model.^11,20^ Nowadays, this has become even more relevant with respect to following the process analytical technology (PAT) framework and quality by design (QbD) principles.

A few systematic attempts for selecting preprocessing utilizing design of experiments^11,21^ and genetic algorithms^22−24^ have been reported in the literature. Yet, the vast majority of the aforementioned methods considers a sequential transition from data preprocessing to model regression and thus fails to capture their synergistic effect, meaning the influence of each step on one another and on the overall model performance.

Another challenge associated with the generation of the regression model itself is the performance criterion upon which the PLS model is built. In general, different criteria will lead to different PLS models.^25^ In most cases, an accuracy metric, commonly root mean square error prediction (RMSEP), is used to evaluate the predictive ability of the model.^20,24,26^ Yet, a model created solely based on the quantitative performance of the prediction is not necessarily interpretable and robust to exogenous variability, especially with respect to new samples. It is therefore crucial for the validity of the model that both accuracy and robustness metrics are taken into account.^12^

The scope and novelty of this manuscript is 2-fold. First, we introduce a robustness metric to handle variability arising from known interferences in the data. Second, we incorporate preprocessing decisions and PLS regression in a single optimization step toward automating and updating the model generation process.

The rest of the manuscript is structured as follows. Section 2 discusses general preprocessing aspects as well as the specific preprocessing steps considered in this work. Section 3 refers to latent variable regression methods and in particular the generation of the PLS regression model and its predictions. Validation metrics for the regression model and how they are typically approached in the literature are further discussed. Then, the formulation of the optimization problem is described in section 4. Section 5 presents illustrative examples using the proposed optimization method, whereas section 6 concludes this work by summarizing the main outcomes and suggesting future directions.

Throughout the manuscript the following notation is adapted. Matrices are indicated with capital bold letters, vectors with lowercase bold letters, and scalars with normal lowercase letters. If not explicitly specified, matrices, vectors, and scalars are expected to have real numbers as entries. All vectors are considered to be column vectors unless stated otherwise. In the subscript, *i* indicates the *i*th row and *j* the *j*th column of a matrix or vector unless stated otherwise. For example, given a (real) matrix **X** with dimensions *n* × *m*, **x**_*i*_ of size *m* × 1 indicates the *i*th row of the matrix and *x*_*ij*_ the element in the *i*th row and *j* the *j*th column of the matrix. It follows that **X** = [**x**_*i*=1_...**x**_*i*=*n*_]^*T*^ = [**x**_*j*=1_... **x**_*j*=*m*_]. A full list of abbreviations and symbols is provided in the nomenclature section in the Supporting Information.

## 2 Data Preprocessing

Data preprocessing may include a variety of steps, such as vector normalization, multiplicative^27^ or orthogonal^28^ signal correction, standard normal variate (SNV),^29^ and Savitzky–Golay (SG)^30^ filters. Some of the existing preprocessing steps are parametric, i.e., they include choices that typically need to be made before they can be applied to the data, and some are not, i.e., they can be directly applied to the data without any prior decision making. Some exemplary listings of parametric vs nonparametric preprocessing are available in work by Jarvis and Goodacre,^22^ Devos and Duponchel,^23^ and Bocklitz et al.^24^ An attempt to summarize these is presented in Table 1. In general, human expertise is used to determine the choice of the preprocessing steps and, when relevant, the choices of their parameters as well as the order of these steps. A more efficient way for making these choices is to include them as decision variables into an optimization problem (see section 4).

**Table 1:** Summary of Some Typical Parametric and Nonparametric Preprocessing Steps Encountered in the Literature^22−24^

type	parametric^a^	nonparametric
background^24^	polynomial fit(1), kernel derivative(1), SNIP algorithm(1),^31^ SG(3)	
		
smoothing and filtering^22−24^	kernel smoothing(1), SG(2), one-dimensional mean filter of order n(1), weighted least-squares baseline of polynomial order(1)	
		
normalization, scaling, and centering^22−24^	lp-norm^b^(1), peak-norm^c^(1)	M–M norm,^d^ M–V norm,^e^ MSC,^f^ normalize to total,^g^ SNV, autoscale, mean-center, median-center, square root mean scale, variance scaling
		
derivatization^22,23^	SG(3), derivatize straight line fit by sliding window of size ws(1)	
		
baseline correction^22^		normalize first bin to zero, subtract average of first and last bin, detrend by subtracting a linearly decreasing baseline

a The numbers in parentheses indicate the number of parameters that the specific preprocessing method requires.

b Equivalent to the maximum norm.^24^

c Normalization on a peak.^24^

d Min–max normalization. Normalize maximum to one and minimum to zero.^22,24^

e Centering to zero mean and scaling to unit variance.^22,24^

f Multiplicative scatter correction.^23^

g Sums equal to unity.^22^

In this manuscript, SNV followed by a SG filter and column-wise mean centering are solely used as preprocessing steps. Consider a data set with the raw input measurements of the training set, **X**_*m*_ of *s* × *p* size, where *s* is the number of samples or observations and *p* the number of frequencies measured by a spectrometer. The mathematical row-wise transformation from applying SNV to the data is presented in Supporting Information SA. Note that the SNV step is nonparametric, and thus, given a certain data set, this process is fully automated in that there are no decision variables associated with it. On the contrary, SG^30^ is a parametric preprocessing method. The decision variables related with it consist of the window size (*ws*), the order of the polynomial (*op*), and the order of the derivative (*od*). Given the input measurements of the training set after vector normalization, **X**_*snv*_ of *s* × *p* size, and the preprocessing parameter vector of positive integers, **ω** = (*ws op od*)^*T*^, SG uniquely defines the preprocessing function *g*_*SG*_(·, **ω**): **X**_*SG*_ = *g*_*SG*_(**X**_*snv*_, **ω**), with **X**_*SG*_ of size *s* × (*p* – 2*ws*) being the input measurements matrix of the training set after vector normalization and application of the SG filter. Column-wise mean centering is also nonparametric, and the exact formulation can be found in Supporting Information SA.

We can define the general preprocessing function for the regressor that includes all SNV step, the SG filter and column-wise mean-centering, *g*(·, **ω**): **X** = *g*(**X**_*m*_, **ω**), where **X** of size *s* × *n* are the input measurements of the training set after preprocessing and *n* is the number of independent variables. Note that the number of columns in **X** differs from **X**_*m*_, due to the SG transform (*n* = *p* – 2*ws*).

The raw output measurements of the training set, **Y**_*m*_ of size *s* × *m*, with *m* the number of dependent variables are only subject to column-wise mean-centering to obtain the preprocessed response matrix **Y** of size *s* × *m* (see Supporting Information SA).

## 3 Latent Variable Regression

In multivariate calibration there are many input variables that can be measured, many of which could be highly correlated. Latent variable regression methods aim at decreasing the dimensionality of the data by projecting them to lower dimensions.^3^ Alternative methods for dimensionality reduction, such as the use of autoencoders,^32^ are possible. In the following, our analysis is constrained to the PLS latent variable regression method. PLS relates an input and an output data set assuming that at least one of the two variable spaces (one associated with each data set) has a reduced rank.^7^ More precisely, in PLS, the principal components or latent variables are chosen to maximize the covariance between the input and the output data.

### 3.1 PLS Model

Consider a regressor, **X** of size *s* × *n*, and a response, **Y** of size *s* × *m*, obtained after the preprocessing described in section 2, defining the training data set. We assume that the examined process is driven by a number of latent variables α ≤ *n*. Then, the PLS model corresponding to the underlying latent structure has the following form
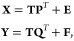
1where **T** the scores matrix has size *s* × α, **P** and **Q** the loadings matrices have size *n* × α and *m* × α, respectively, **E** the random error in the measurements of **X** has size *s* × *n*, and **F** the random error in the measurements of **Y** has size *s* × *m*.

The PLS model described by System 1 can be obtained by using NIPALS algorithm. More information about the algorithm and the intermediate steps and matrices can be found in the Supporting Information SA. For the scope of this work, we describe NIPALS algorithm as *h*_NIPALS_(·,·,·), a function that given **X**, **Y**, α will return the weights matrix **W** of size *n* × α as well as the loadings matrices **P**, **Q**, such that the following system of equations holds
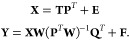
2

### 3.2 Model Prediction

Let us now assume new raw measurement regressor and response matrices constituting the validation set **X**_*m*_^′^ of size *o* × *p* and **Y**_*m*_^′^ of size *o* × *m*, respectively, with *o* being the number of samples or observations in the validation set. By applying data preprocessing as in section 2 and utilizing the PLS model developed in section 3.1, we can obtain the reconstruction/prediction of **X**′, being **X̂**′ of size *o* × *n*, and the prediction of **Y**′, being **Ŷ**′ of size *o* × *m*. Note again that *n* = *p* – 2*ws* due to the SG transform. Detailed information about the derivation of these prediction matrices is provided in Supporting Information SA.

### 3.3 Validation Metrics

A range of validation metrics to quantify the performance of a regression model in pharmaceutical applications can be found in the European Medicines Agency (EMA)^33^ and United States Food and Drug Administration (FDA)^34^ guidelines. These include, among others, model accuracy, precision, and robustness. The International Council for Harmonisation of Technical Requirements for Registration of Pharmaceuticals for Human Use (ICH) guidelines^35^ in an attempt to promote pharmaceutical development and manufacturing provide some conceptual definitions for these metrics.^36^ Further desired characteristics and suggestions for their practical consideration in real-world applications have also been proposed in these guidelines. In this section, we attempt to summarize the main points and provide a brief overview on how these metrics have been utilized so far in the literature.

Model accuracy is typically put forward in terms of goodness of fit of the prediction. As previously mentioned, the use of RMSEP is commonly utilized in this regard.^37,38^ Alternative/complementary metrics for accuracy, such as root-mean-square error of calibration, or cross validation and/or bias are conceivable. These measures are sufficient for understanding the average errors in a data set; however they should not be interpreted as an estimate for the uncertainty of a specific prediction at a new input.^39^ Moreover, use of these measures ignores the fact that errors can come from different distributions that have the same mean error but different shapes.^40^

Precision can be measured with repeatability, i.e., standard deviation of repeated measurements of the same sample, where the sample is not moved during spectral acquisition.^35^ Precision can be extended to intermediate precision and reproducibility by accounting for variation within and across laboratories, respectively.^35^ It is clear that a model can be precise but not accurate, for example, in cases where many experiments performed under identical conditions give data that predict similar model outputs, yet those predictions are far off from the true/reference value of the output.

The ICH guidance^35^ refers to robustness as the ability of the predictions to not be affected by variations that occur in manufacturing and environmental conditions. In that sense, robustness can be seen as expanding the idea of precision to variations in the production process, such as scale of the plant, dosage of tablets, etc. This consideration of robustness is often brought up in the literature, see, e.g., work by Wise and Roginski,^41^ Hetrick et al.,^42^ Ferreira et al.,^43^ Vargas et al.,^44^ and Muñoz and Torres.^45^ A different consideration of robustness is the statistical one,^32,46−48^ which focuses on outliers detection. Note that in both the EMA^33^ and the FDA^34^ guidelines rejecting outliers is addressed in the context of another metric, specificity, and is not further discussed here. Moreover, within the broader context of the increasing efforts toward continuous manufacturing, robustness of a model can be also associated with obtaining good predictions for a prolonged period of time, i.e., increasing up-time of a model.^37^ Although this idea of prolonging model applicability extends the concept of robustness to include the time dimension, it does not touch upon the definition of robustness at the time of model creation, and thus, it is not further discussed here.

The remainder of this discussion focuses on exploring the trade-offs between accuracy and robustness and quantifying the robustness metric.

As previously stated, an accuracy metric tries to capture the difference from a reference or true value.^35^ Therefore, regression models built on this metric are often overparametrized, i.e., they tend to require a higher number of latent variables than the one that is actually needed. This leads to fitting unnecessary noise in the data, and although it does not cause significant problems initially, the performance of the regression to new data may deteriorate quickly. In practice, heuristic metrics, such as percentage of improvement to the accuracy metric by adding another latent variable, are most of the time used to decide the number of latent variables of a regression model targeting accuracy.

With respect to robustness, for the scope of this work, we are interested in achieving model insensitivity to known sources of variability that are not related to the active pharmaceutical ingredient (API), for example, the production scale. Robustness in this concept is often checked a posteriori in the literature, see, e.g., work by Vargas et al.,^44^ Kamyar et al.,^49^ and Sierra-Vega et al.^50^ FDA guidance^34^ suggests that robustness can be implicitly accounted for during the model calibration step by incorporating all expected sources of variability. By including all variation, it is anticipated that a regression model with the appropriate number of latent variables would not respond to unwanted sources of variability in the input. In this direction, also multimodel approaches that can split the known variability realizations into clusters are conceivable.^51,52^ However, approaches including all variations in the data can be generally impractical, as they may require very large data sets, which can increase experimental efforts.^53^ To avoid this problem, design of experiments for the calibration set to obtain maximum information on the variation has been also investigated in the literature, e.g., Alam et al.^38^ and Li et al.^54^ Furthermore, sometimes incorporating all variations might not be possible, as it may require data from the future.^53^ Such an example may for instance occur when there is data available for a process on lab and pilot scale, but we are interested in building a model that performs well in a fully industrial scale, although we still do not have any data for this scale beforehand. Another example would be building a model on data collected from currently available instruments, where in a future scenario when an instrument becomes defective we can replace it with a new one with minimal model maintenance efforts. One approach to account for this is developing a model that focuses on maximizing information from the signal of the analyte of interest.^53^ Orthogonalization methods^55−57^ aim at removing information not related to the signal of interest. However, such approaches do not take into account information about the sources of variation that are already known and thus could be useful to incorporate.

In the next section, we propose a mathematical definition of robustness based on moment matching that explicitly accounts for known variability sources in the data and tries to maximize insensitivity of the prediction to such variations. Our approach is constrained to the use of a single model for the whole set of data, as this is often favored from an industrial perspective due to simplicity and ease of use. Particularly when considering model maintenance, this becomes of high importance. Overall, models built on robustness are anticipated in the literature to give simpler models, i.e., models with a fewer number of latent variables, that might not initially give the most accurate predictions but are in general less amenable to degradation with time. This is crucial as it can decrease the frequency and effort for model updates and maintenance and is in alliance with what was reported in Alam et al.^38^

## 4 Optimization Problem Formulation

As discussed previously (see section 1), there are many decisions associated with data preprocessing and model regression that are often selected on an ad-hoc basis by experts. Optimization methods for utilizing the high data availability from spectroscopic measurements to obtain process knowledge have been used previously, e.g., kinetic parameter estimation.^58−60^ Additionally, an optimization framework for model-based experimental design to advance process understanding in compliance with the QbD paradigm is presented in Chen et al.^61^ This section is devoted in breaking down and defining the main parts of an optimization problem that can provide an alternative, systematic approach for decision making in building and maintaining chemometric models.

### 4.1 Objective Function

Among the challenges of creating a predictive PLS model is the identification of the overall objectives for the application being built. Most attempts in this direction focus in creating a model that provides an accurate model prediction. However, this is often not sufficient since the model is typically subject to known sources of variability against which we want the model to be robust. In this regard, detailed definitions for both accuracy and robustness are provided below.

#### 4.1.1 Accuracy

Given a validation set with output measurements **Y**′ and the predictions obtained by the model **Ŷ**′ (both mean centered), the square prediction error, *spe*, can be used as an indicator for accuracy
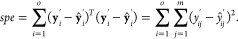
We can define the root mean squared error of the predictions (*RMSEP*) as
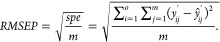
Note that *RMSEP* is in practice easier to interpret (because it uses raw units), and thus, in the following it is used as an optimization criterion for accuracy.

#### 4.1.2 Robustness

For simplicity, let us assume one known source of variability with *n*_*l*_ being the number of possible realizations (positive integer). For example, if we consider the scale of a plant as the known source of variability and assume that the measurements were taken at three different scales, A, B, and C, then in this case *n*_*l*_ = 3. We define vector **z** of size *s* × 1, where each element, *z*_*i*_, provides information about which of the *l* = 1, ..., *n*_*l*_ possible realizations is true during the *i*th observation. For example, *z*_*i*_ = 1 for that observation could indicate that the corresponding measurement was taken in scale A.

Our intuition for the robustness metric is the following. We would like the model to behave identical regardless of which realization of a variability source is true. If this is the case, we would expect similar distributions of predictions from the model for similar sets of training data. One way of measuring the similarity of different distributions is to compare their mean and variance, which is the method of matching moments.^62^ Note that we anticipate the summary statistics of the mean and the variance to be sufficient, as the distribution of the output should be unimodal for the applications of spectroscopy that we are interested in.

In particular, to define robustness, we consider the method of moments and we attempt matching of the first and second sample moments across all different realizations of the known variability in the considered PLS model. The first moment refers to the sample mean and the second one to the sample variance. In our scale example, this includes first subdividing the training set on three subsets, depending on which of the three scales was active in each measurement, then calculating the mean and variance of each subset, and finally minimizing the (pairwise) distances in the mean (first moment) and the variance (second moment) across all three subsets.

Note that for the calculation of our robustness metric, the number of measurements within each realization of a variability source does not need to be equal. However, the samples particularly for unrepresented categories need to be representative. Note also that we assume similar concentration levels from the samples across the different categories for better explainability of the metric.

In general, we do not necessarily need complete knowledge of all possible distinct values of a known variability to apply our robustness definition. As we will also illustrate with the example in section 5.1, even in cases that only a few values are known, we can create a model that is overall robust to the considered variability source. Continuous variation is not considered so far with our approach; however, it can be easily extended to account for it in cases where some kind of clustering is applicable.

**Figure 1:**
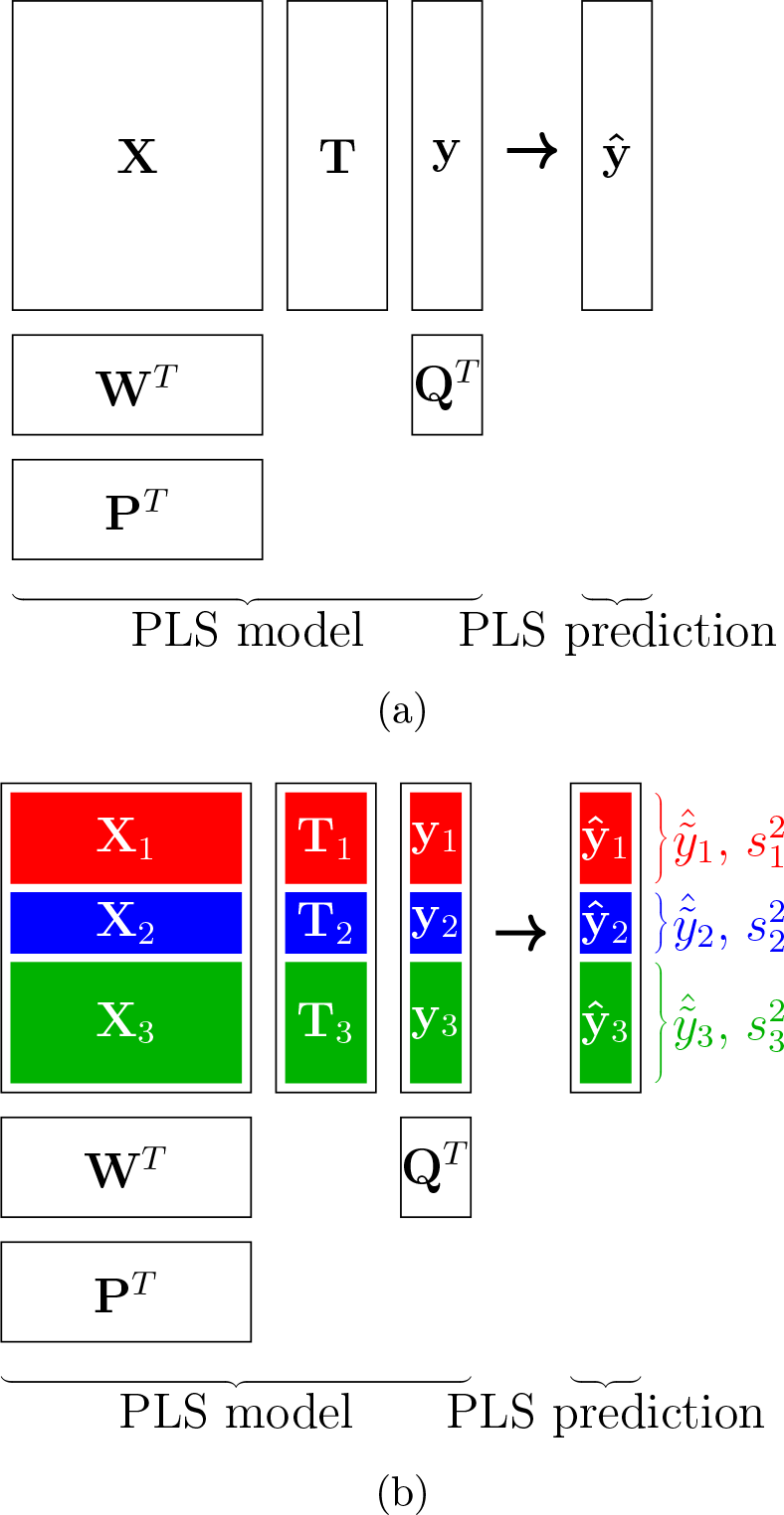
Comparison of (a) plain PLS vs (b) PLS with the robustness metric in matrix representations for one known source of variability in the data. The considered variability source here has three different realizations that are depicted by the three different colors. The average prediction, , and the variance, *s*^2^, for each realization are used for the quantification of the robustness metric.

**Figure 2:**
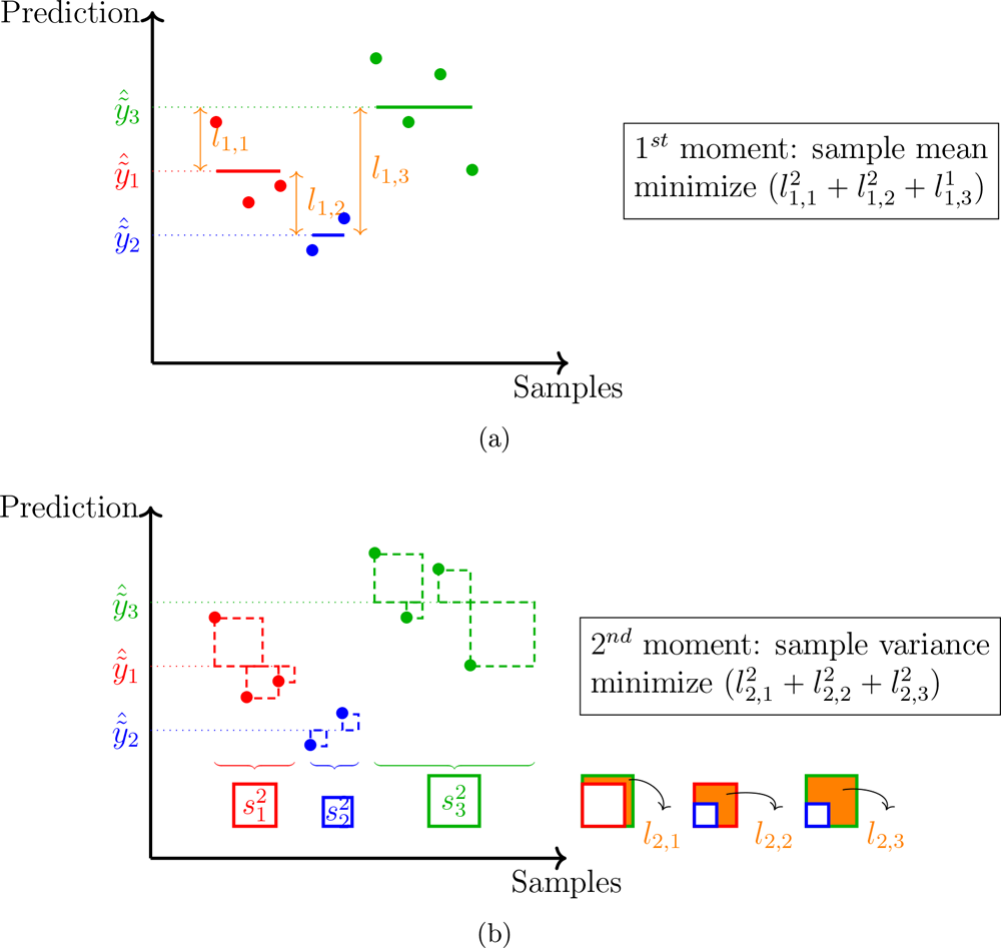
First and second moment representations for one source of variability with three different realizations depicted by the three different colors. The average prediction, , and the variance, *s*^2^, for each realization are the ones obtained from Figure 1.

In Figure 1, we provide a graphical representation of the robustness metric and its comparison to a plain PLS model. In particular, the black boxes represent the PLS matrices, as they are described in section 3.1. For illustration purposes, a one-dimensional output, **y**, is considered. Figure 1 is tailored to the scale example with one source of variability and three different realizations. In other words, the three different colors in Figure 1 represent the observations from the three different scales. The PLS matrices are then used together with the corresponding subset of the input matrix (colored boxes in **X**) to obtain the model prediction for the output, **ŷ**. The mean, , and the variance, *s*^2^, for each subset of those predictions, i.e., the different colored boxes in **ŷ**, are then calculated. The minimization of the distances for both the mean, see Figure 2a, and the variance, see Figure 2b, across those subsets in **ŷ** composes the objective of the robustness metric.

Note that we assume that the different realizations are uncorrelated with other sources of variations that affect the inputs; thus, we expect this metric to be more effective in cases where this assumption holds.

To formalize this procedure, we first define the subsets of input, **X**_*l*_ of size *s*_*l*_ × *n*, and output, **Y**_*l*_ of size *s*_*l*_ × *m*, measurements that correspond to the *l*th realization of a given variability source, **X**_*l*_, **Y**_*l*_ ⊂ **X**, **Y**: **x**_*tr*,*i*_, **y**_*tr, i*_ ∈ **X**_*l*_, **Y**_*l*_ ⇔ *z*_*i*_ = *l*, ∀ *i* ∈ [1,...,*s*], ∀ *l* ∈ [1, ..., *n*_*l*_], with ∑_*l* = 1_^*n*_*l*_^*s*_*l*_ = *s*. For the moment matching, the squared (Euclidean) distances among the *n*_*l*_ different realizations are calculated, and their sum should be minimized. More precisely, given the first and second moments, **L**_1_,  distances are evaluated, where . The total distance for each moment is
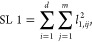

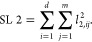


We can define the robustness (moment matching) objective as
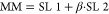
3where β is a weight factor between the two moments utilized as relevant to account for differences in the order of magnitudes between the two moments. Of course, β can also be used to place specific emphasis to one of the two moments (see discussion in section 5). Note that alternatively to eq 3, we could have 0 < β < 1 and then have β multiplied by the first moment and (1 – β) multiplied by the second moment. Although this would make no difference to the solution, it could potentially help scaling the two moments. When the differences among average predictions and variances for the considered realizations are small, Manhattan distances may be preferred instead.

In the case of multiple known sources of variability, this procedure is repeated for each individual source and then the average sum of these distances (divided by all number of distances, number of possible realizations for each source, and number of the sources) for each moment is considered. In this case, there is when relevant the additional option to weight the contributions from the different sources.

There is an interesting link between the robustness objective that we have just described and the accuracy metric. It is well known that the accuracy can be decomposed into a bias and a variance term.^63^ The bias of a model for a set of points is given by the mean of the predicted values minus the mean of the true values so is simply the first moment plus a constant. It is already considered good practice to consider biases when selecting models, and we would expect that practitioners who do so would reach similar conclusions to our robustness metric using only the first moment. The second moment that we describe above is also closely related to the variance of the prediction. Thus, in order to fully recover our robustness metric, a practitioner would also need to check that the variances of the predictions remained stable across different realizations of a variability source. The difference between the robustness and the accuracy metric is that the accuracy metric allows trade-offs between the variance and the bias across different manufacturing conditions to minimize the total error, while the robustness metric requires that both the bias and the variance of the predictions remain similar between all realizations of a variability source.

We claim that our robustness metric leads intrinsically to simpler models in the sense of fewer latent variables. In order to see this, note that each new latent variable in the PLS model will look for sources of variability in the input data to improve the prediction. This new source of variability for PLS could be either the source of variability that we would like the model to be robust to or a different source. If the new source of variability is independent from the known variation we want to be robust to then there is a greater than one-half chance that the robustness metric is made worse by an additional latent variable (see Supporting Information SB for proof). In other words, models which describe sources of variability that are independent to the signal we wish to be robust to are likely to have worse robustness objectives. The robustness metric worsening will lead to models with fewer latent variables. It is important to note that although our definition of robustness tends toward decreasing the number of latent variables, it will not always terminate with the smallest number of latent variables available. In particular, if the source of interference affects the output, the moment matching will improve the prediction by bringing those together, and thus, additional components will be added.

### 4.2 Decision Variables

As the choices in data preprocessing affect the PLS regression, decisions commonly taken sequentially for each step are suboptimal. This can be mitigated by incorporating their corresponding variables as decision variables in an optimization problem.

From the discussion in section 2, it is clear that a significant part the decision variables pertains to data preprocessing. Data preprocessing for the scope of this report and unless stated otherwise is set equivalent to SNV followed by a single SG filter and column-wise mean centering (see section 2); thus, the decision variables for any potential optimization in the preprocessing narrow down to defining **ω** = (*ws op od*)^*T*^.

Generation of the regression model for accurate data description and future predictions is also not decision free. In section 3.1, we assumed a number of latent variables α for the PLS model. However, this number is not typically known a priori. In general, different heuristic criteria can be utilized for this scope (e.g., *R*^2^, *AIC*, *spe*).^25,64^ In an optimization setting, to obtain the regression function (as shown in section 3.1), α can be considered as an additional decision variable.

Consequently, given a data set of measurements and a data set of dependent variables (these can be further divided into training and validation sets), potential decision variables for optimization belong in the parameter vector **u**^*T*^ = (**ω**^*T*^ α) = (*ws op od α*).

### 4.3 Problem Definition

Taking all of the previous factors into account, we can now formulate the optimization problem.

For simplicity, let us generalize the previously defined NIPALS function to include also mean centering in the response obtaining function *h*(·,·,·), which given **X**, **Y**_*m*_, α will return **W**, **P**, **Q**.

The objective function for accuracy can be described as *f*_acc_(·,·,·,·,·), a function of **X**_*m*_, **Y**_*m*_, **X**_*m*_^′^, **Y**_*m*_^′^, **u**. Function *f*_acc_ has a real-valued output. Similarly, we can define the objective function associated with robustness as *f*_rob_(·,·,·,·), which given **X**_*m*_, **Y**_*m*_, **u**, β will return a real value output.

The optimization problem for accuracy and robustness becomes
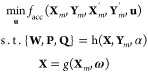
4
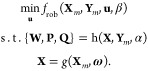
5

## 5 Illustrative Examples

In this section, we test our proposed approach using three illustrative examples from spectroscopy. In all cases examined, three sets of data are considered, namely, a training set that is used to fit the PLS parameters, a validation set that is used for simulating the model building procedure and decide about tuning parameters and model structure, and a test set referring to previously unseen data to provide an unbiased evaluation of the model performance in real-life conditions. The accuracy and robustness objectives are calculated on the validation set. PLS with NIPALS algorithm is executed using the pyphi package^65^ with the mean-centering option on unless stated otherwise. The code is available at https://github.com/ckappatou/AcRoPLS. For the third example, the code is not included for confidentiality reasons. The optimizations are performed in ENTMOOT^66^ with 90 initial points, 200 iterations, and default settings unless stated otherwise. The permissible domain for the decision variables is described by the elements of the following sets ({10, 15, ..., 40}{1, 2, ..., 4}{0, 1, 2}{1, 2, ..., 8})^*T*^ based on our experience and common values. This includes the preprocessing parameters and the number of latent variables for the PLS model. An additional hard constraint was introduced that requires *od* to be lower or equal to *op* for all cases examined for a meaningful data transformation by applying the SG filter. Note that the step size of five in the SG window size is introduced to facilitate comparison of the optimization results to complete enumeration within the considered domain, see discussion in sections 5.2 and 5.3. Complete enumeration serves here as a validation tool to ensure successful termination of the ENTMOOT solver. This would be otherwise difficult to confirm since black-box optimization solvers aka Bayesian optimization solvers are only asymptotically complete,^67^ that is, they can only guarantee to find the global minimizer (if it exists) if the solver runs indefinitely. Using an optimization solver such as ENTMOOT is superior to complete enumeration as the number of decision variables and/or their permissible domain increases. Nevertheless, our method is not limited by any specific solver; thus, instead of ENTMOOT, we could have used any other global black-box optimization solver capable of handling integer or categorical input variables, for example, SMAC3^68^ or SKOPT.^69^

### 5.1 Initial Example

**Figure 3:**
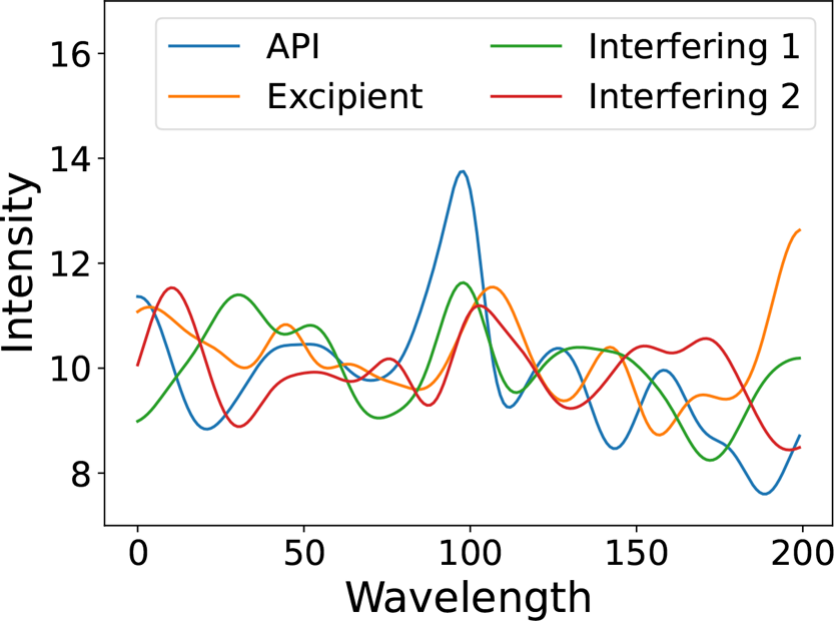
Initial example. Spectral signals for the API, the excipient, and the two interferences; the interfering 1 signal represents the known source of variability and interfering signal 2 the unknown.

We consider the following simulated set up with one API signal, one excipient, and two sources of variability (interfering signals). The spectral signals for this example are illustrated in Figure 3. The one source of variability is assumed to be known, while the other one represents some unknown variability. The known variability source has three different realizations (classes). The relation between the data of the three categories is built by adding a linear component to the API/excipient signal with a categorical element, depending on which category the data point comes from. We test the model performance for both robustness to the known variability source and accuracy.

**Figure 4:**
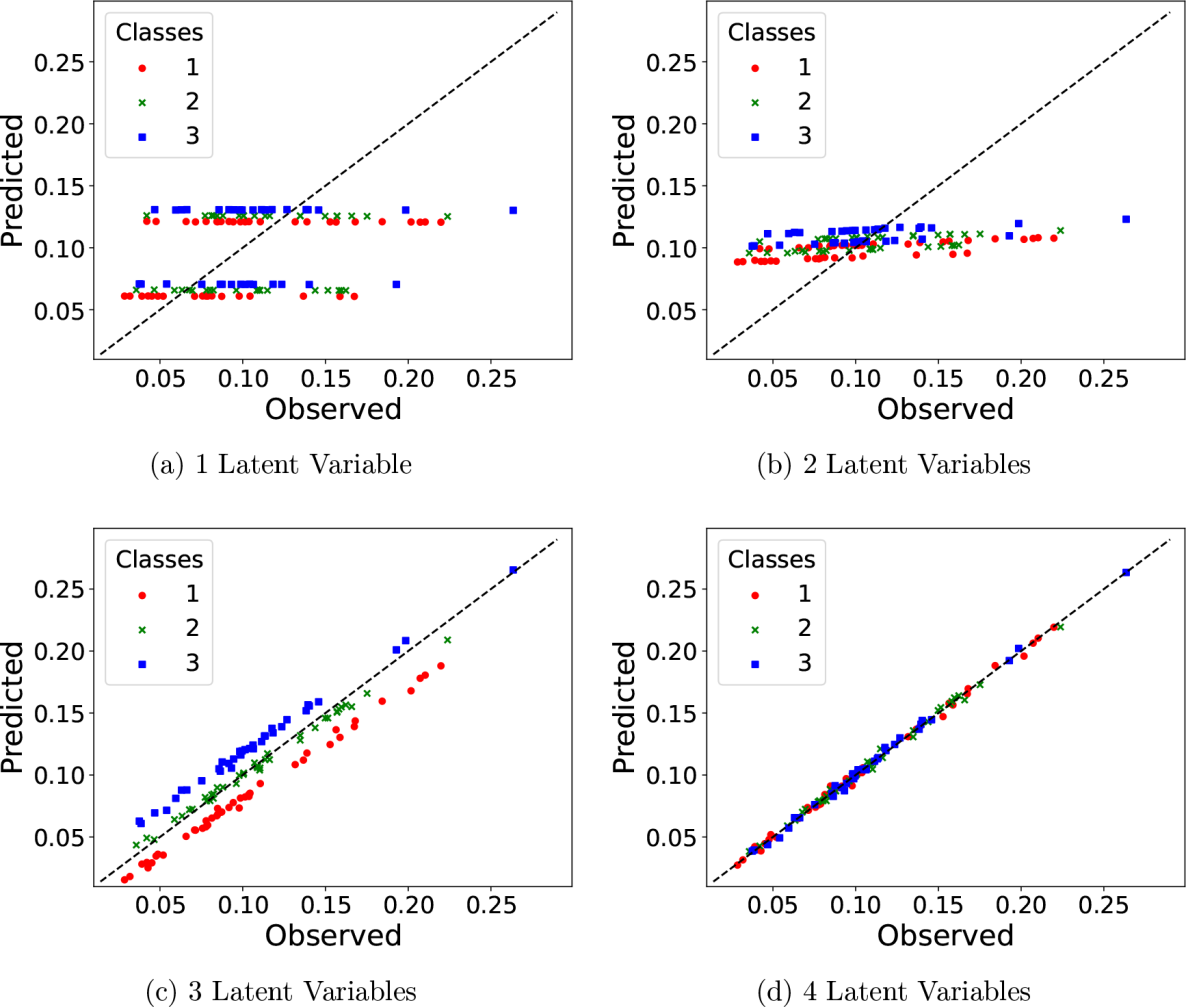
Initial example. Predictive vs observed results for models w/o preprocessing and different numbers of latent variables depicted for the complete data set; different markers/colors indicate different realizations of the known variability source.

For simplicity, let us first examine model building without any data preprocessing. Figure 4 illustrates how models with different numbers of latent variables perform in predicting the API signal. We see that as we keep adding components (latent variables) the prediction improves. By having a model with four latent variables, we obtain good agreement between predicted vs observed values. This is reasonable as most variation in the data (except some random noise) comes from four sources, namely, the API signal, the excipient signal, and the two variability sources.

**Figure 5:**
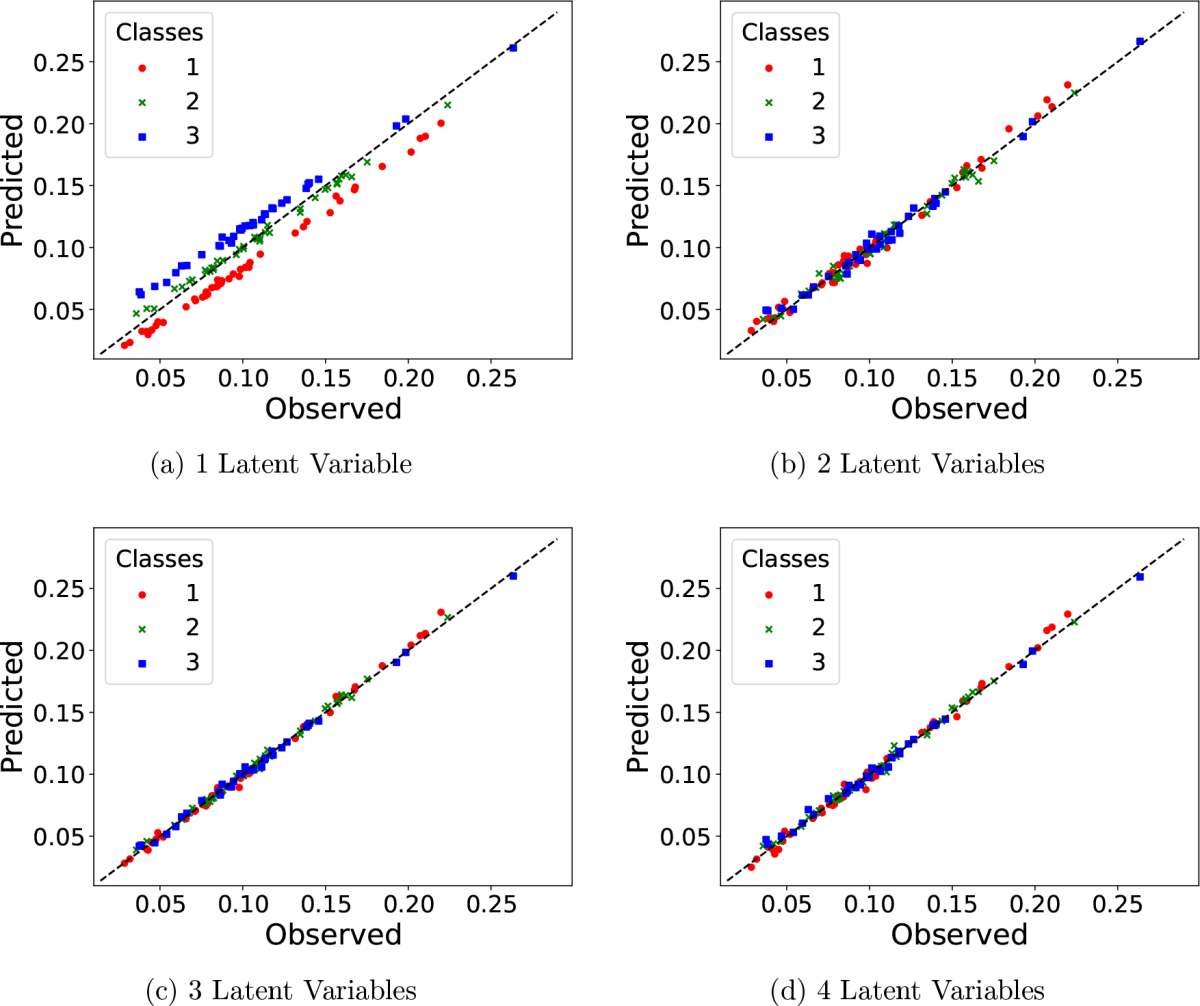
Initial example. Predictive vs observed results for models with nonparametric preprocessing and different numbers of latent variables depicted for the complete data set; different markers/colors indicate different realizations of the known variability source.

To gain some insight on the effect that the preprocessing steps can have on the regression model, let us now assume a simple nonparametric preprocessing including SNV and mean centering (see Supporting Information SA). The performance of different regression models (different numbers of latent variables in each one) is shown in Figure 5. We observe that in this case already a two latent variable model does a good job in predicting process behavior. This is a simple yet powerful illustration of how preprocessing affects model building. It also indicates how choosing appropriate preprocessing can reduce model complexity; in this case, we need a model with one-half the number of latent variables than before to achieve similar model performance.

**Figure 6:**
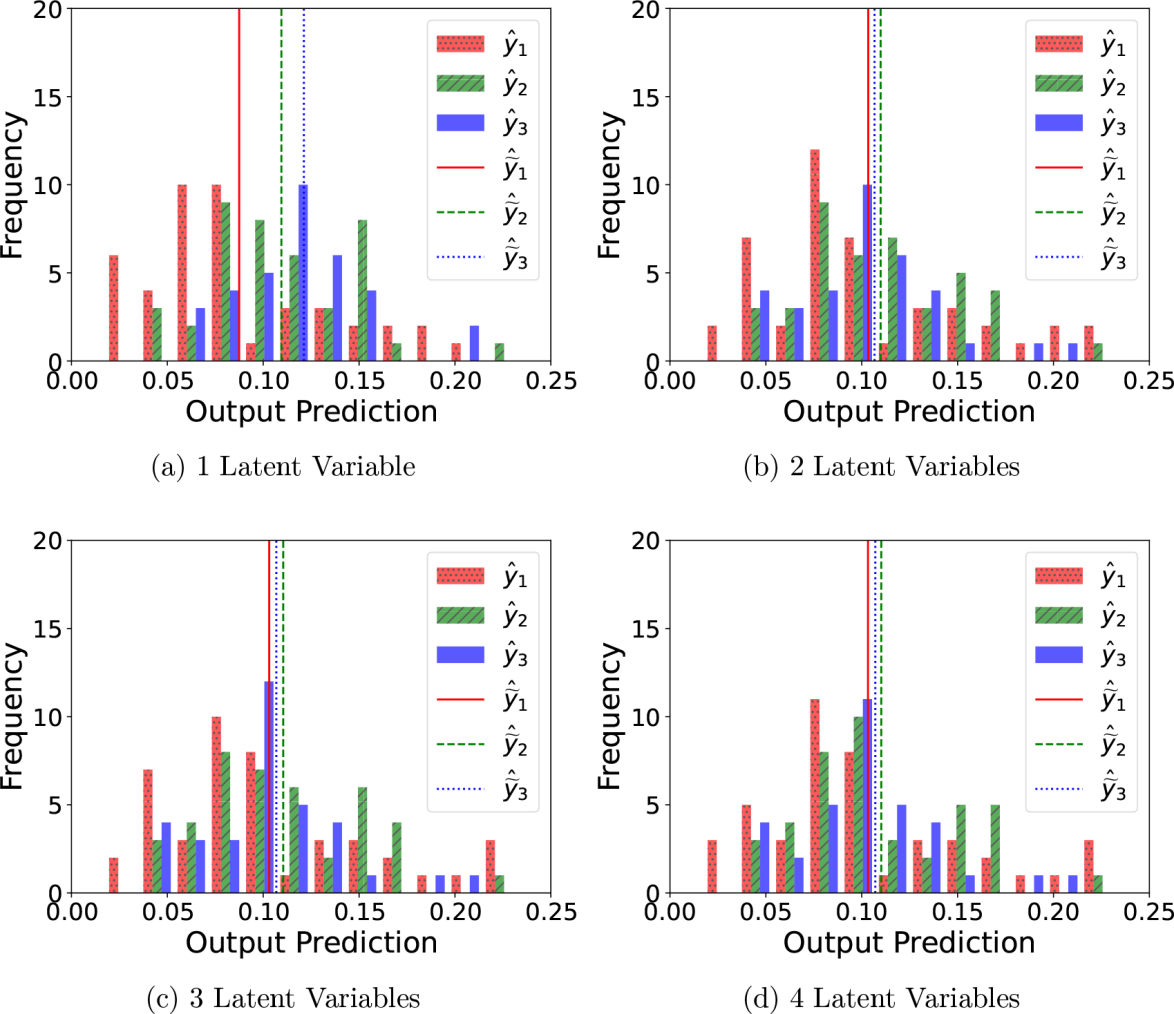
Initial example. Histograms with distributions of the output *y* for models with different numbers of latent variables depicted for the complete data set; different colors indicate different realizations of the known variability source and , ,  the prediction of the means of the distribution of each realizations of the known variability source.

However, deciding when to stop adding components (latent variables) to avoid overfitting is in general not intrinsic. We claim that our moment matching definition can serve as a useful tool in this regard. Figure 6 shows the histogram of the distributions with the different models. The solid lines represent the means of the different realizations of the known variability source, and thus, the distances between those lines provide an illustration on how the first moment in our moment matching objective behaves with an increasing number of latent variables. From Figure 6 is clear that a model with two latent variables has the smallest distances across the different classes and thus would be selected from our moment matching definition.

To be able to quantify overfitting, let us now assume that only data from two out of three realizations of the known variability source is available. To make sure we are not interpolating, it is important that one of the two selected realizations is the middle one. We consider again the same nonparametric preprocessing as before, and we build different models by changing the number of latent variables each time. We split the data set with the two realizations that we consider to 70% training and the rest the validation set. As test set we use the data from the third realization of the known variability. We observe the model performance in predicting both the validation and the test data sets.

**Figure 7:**
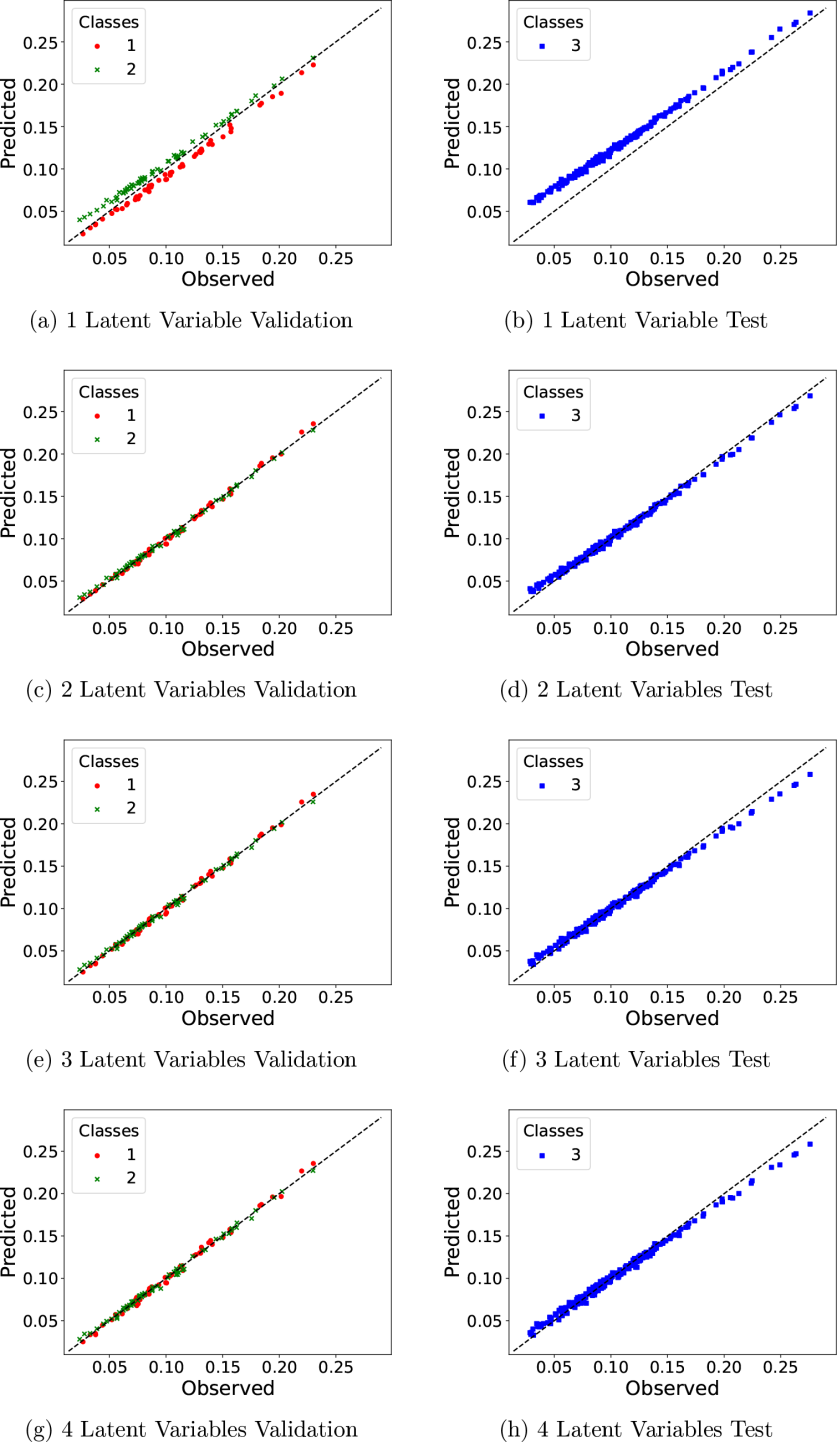
Initial example. Predictive vs observed results for models with nonparametric preprocessing and different numbers of latent variables depicted for the validation data set and the test set; different colors indicate different realizations of the known variability source.

The results for models built with different numbers of latent variables are shown in Figure 7. It is obvious that a model with two components (which is the one our moment matching definition would favor) is the most robust in that it gives a good prediction even for data from a variability realization that was not included in model building. This can be seen clearly on the far right points of the test sets in Figure 7. After this point, continuing adding latent variables leads to an increasing model overfitting effect.

Let us now expand the preprocessing options by including also the SG filter. We first split the whole original data set into 70% training, 20% validation, and 10% test set. We then perform optimizations including both SG parameters and the number of latent variables as decision variables (see section 4.2) for both the accuracy and the robustness objectives. For the robustness definition, β is set to 15 for an even contribution between the moments.

**Figure 8:**
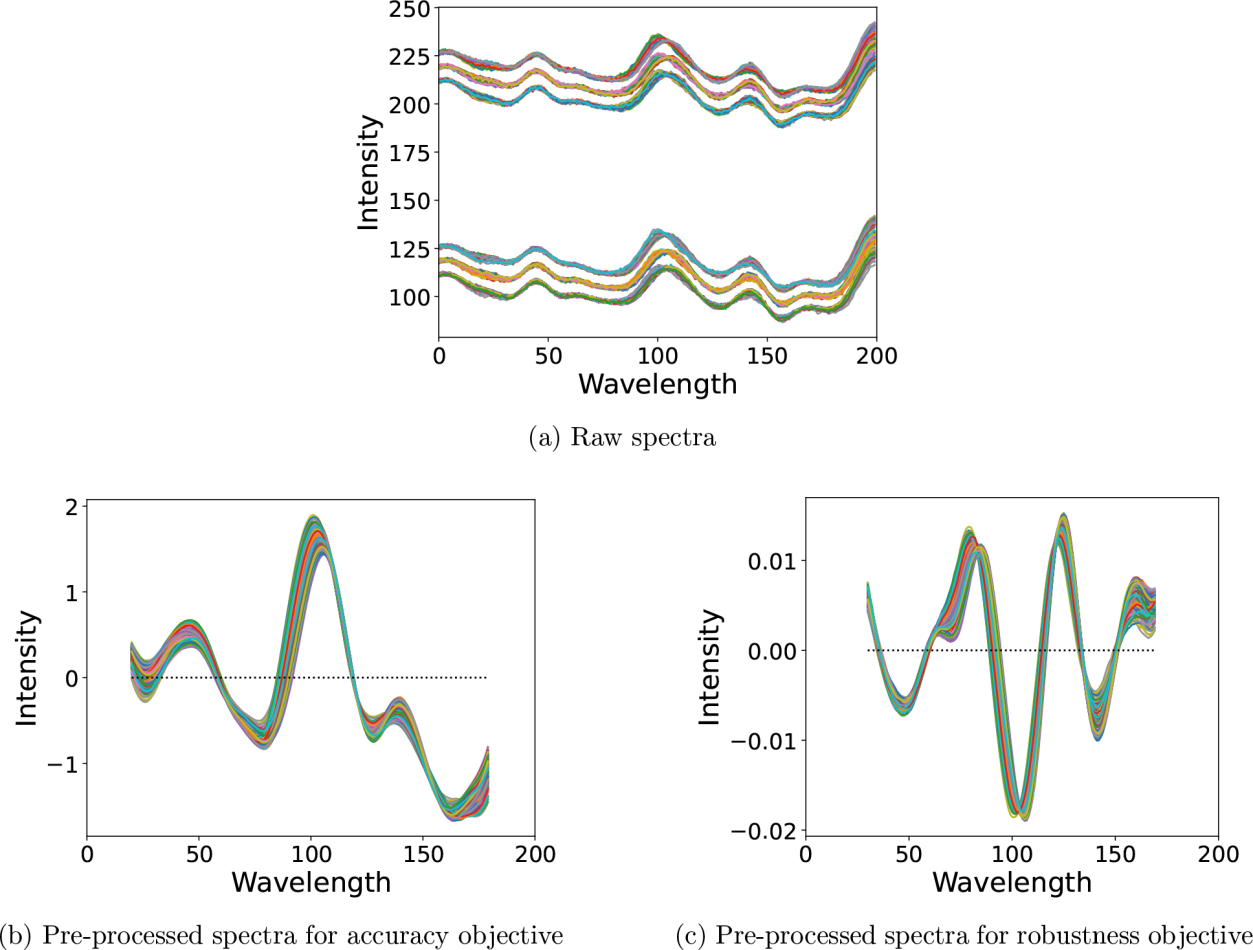
Initial example. (a) Raw and (b and c) preprocessed spectra depicted for the training data set using optimal preprocessing from optimization with accuracy and robustness objectives, respectively

Table 2 summarizes the optimization results. Notice that with the accuracy objective on the validation set the optimization terminates at three latent variables, while optimization with our robustness metric terminates at two. As we showed in Figure 7, addition of the extra latent variable in the case of the accuracy objectives is amenable to overfitting. On the contrary, our moment matching objective was able to track and terminate to the most robust solution for this particular variability source. The raw spectra as well as the spectra after SNV and SG transform for both accuracy and robustness objectives are provided in Figure 8. In Figure 8a, the two discrete bands (upper half and lower half of the figure) derive from the unknown source of variability, while within each of these two bands the separation to three additional bands occurs due to the known source of variability considered here. We observe that as expected the accuracy objective selects a preprocessing that puts the main emphasis on the area of the spectra that makes the API more visible. This is not necessarily the case for the robustness objective. Overall, the low absolute values of intensities achieved when optimizing for robustness are in alignment with the principle of removing unwanted variability in the data, thus leading to derivation of more robust models.

**Table 2:** Numerical Results for Optimization of Data Preprocessing and PLS Regression Using Accuracy and Robustness Objectives in the Initial Example^a^

objective	Dec. Var.	*RMSEP*	MM
accuracy	(20 2 0 3)	0.281 × 10^–2^	0.982 × 10^–2^
robustness	(30 4 2 2)	0.514 × 10^–2^	0.608 × 10^–2^

a Dec. Var.: decision variables **u**^*T*^ = (*ws op od α*). *RMSEP*: root mean squared error prediction in the validation set. *MM*: moment matching as sum of first and second moment squared L1 distances in the training set.

### 5.2 Example from NIR and Raman Spectroscopy

As a second case study, we consider an example from NIR and Raman spectroscopy, originally presented in work by Dyrby et al.^26^ The authors^26^ discuss the development of a PLS model to predict the content of the API in pharmaceutical tablets with two different concentrations, four dosages, and three different scales. The results showed that NIR transmittance is more appropriate for the quantitative qualification of the API. However, the considerable diversity of the examined tablet characteristics poses significant challenges to the samples calibration overall.^26^

We first split the considered data set into 70% training, 20% validation, and 10% test. We then perform optimizations with both objectives. Robustness with respect to scale, dosages, and both known sources of variability is considered. For the robustness objective, β is set by trial and error to 0.005 to obtain a balanced contribution between the two moments. Additionally, a weight equal to two was introduced for the robustness with respect to scale.

**Figure 9:**
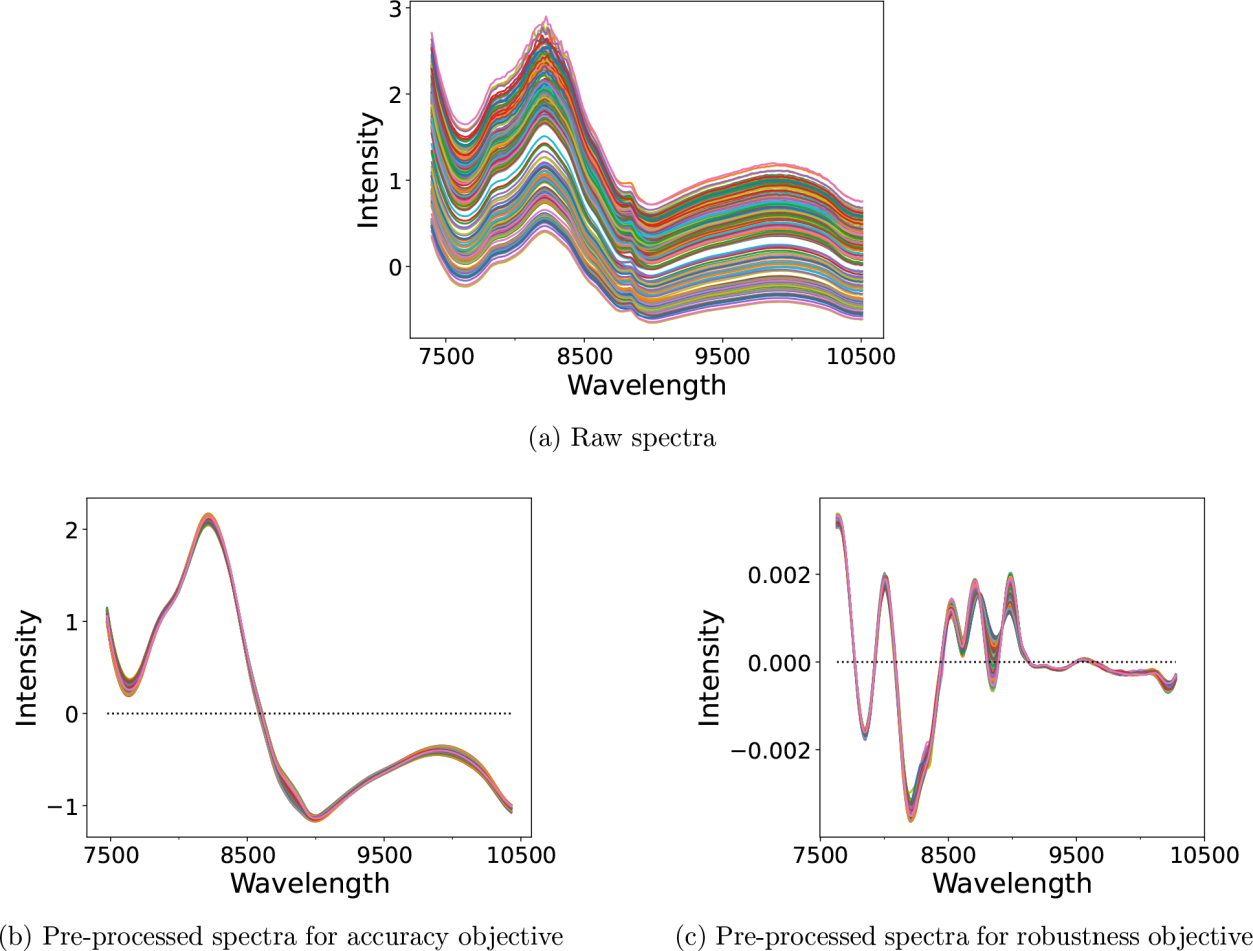
NIR and Raman spectroscopy example. (a) Raw and (b and c) preprocessed (after SNV plus SG transform) spectra depicted for the training data set using optimal preprocessing from optimization with accuracy and robustness with all known sources of variation, respectively.

The optimization results are shown in Table 3. We observe that optimization for accuracy leads to a high number of latent variables, namely, seven, which has an increased risk for model overfitting. Optimizations with robustness have in general lower numbers of latent variables but with the cost of higher *RMSEP* values. Interestingly, the models built for robustness on scale or both known variability sources yield low model complexity, i.e., includes only one latent variable, while still maintaining good performance in terms of model accuracy. Figure 9 depicts the raw and preprocessed (SNV plus SG) results based on the accuracy and robustness with all known sources of variability objectives spectra. Again, the accuracy objective seems to focus on one main peak that probably attempts to best explain the API, while the robustness objective is not constrained to only this region. Also, here the robustness objective seems to be removing unwanted variability from the data that we would anticipate to be part of the known sources of variability we want the model to be robust against.

**Table 3:** Numerical Results for Optimization of Data Preprocessing and PLS Regression Using Accuracy and Robustness Objectives in the NIR and Raman Spectroscopy Example^a^

objective	Dec. Var.	*RMSEP*	*RMSEP_t_*
accuracy	(10 1 0 7)	0.235	0.327
robustness (scale)	(25 3 2 1)	0.279	0.365
robustness (dosage)	(40 1 0 1)	0.677	0.746
robustness (both)	(30 4 2 1)	0.289	0.389

a Dec. Var.: decision variables **u**^*T*^ = (*ws op od α*). *RMSEP*: root mean squared error prediction in the validation set. *RMSEP*_*t*_: root mean squared error prediction in the test set.

The fact that for all cases examined there is no significant discrepancy between the *RMSEP* and the *RMSEP*_*t*_ values is indicative of the homogeneity of the training, validation, and test sets (in this case, all obtained with random split from the original data set).

Complete enumeration within the allowed decision domain (see first paragraph in section 5) was additionally performed for all of the above cases, with the best solution being identical with the one obtained from our optimizations for three out of four cases and with a difference of less than 0.1% for the fourth one. This indicates a sufficient number of iterations and initial guesses for obtaining the global solution.

As these models are purely data driven, it is interesting to examine their sensitivity to the given data. For this, five different random splits (maintaining the 70–20–10% training vs validation vs test ratio constant) are analyzed. Split number one is the one reported so far.

The results are presented in Table 4. We observe that in general accuracy models point toward a high number of latent variables and on average smaller window sizes, while robustness models mostly are the other way around, i.e., they have a small number of latent variables and larger window sizes. No particular trend for the polynomial and derivative order can be identified, as these seem to be data specific. Overall, robustness models would be expected to be less sensitive to data variability; however, in this example, this is only clearly visible in the case of robustness with respect to dosage. This can be due to multiplicity of global solutions or the small number of random splits considered here.

**Table 4:** Numerical Results for Optimization of Data Preprocessing and PLS Regression Using Different Random Splits for the Different Objectives in the NIR and Raman Spectroscopy Example^a^

objective	random 1	random 2	random 3	random 4	random 5
					
**accuracy**					
Dec. Var.	(10 1 0 7)	(30 3 1 4)	(15 2 1 7)	(40 4 0 8)	(15 2 0 8)
*RMSEP*	0.235	0.266	0.299	0.299	0.283
					
**robustness (scale)**					
Dec. Var.	(25 3 2 1)	(30 1 1 2)	(40 1 1 3)	(35 0 1 5)	(30 3 2 2)
*RMSEP*	0.279	0.208	0.346	0.314	0.337
					
**robustness (dosage)**					
Dec. Var.	(40 1 0 1)	(40 1 0 1)	(40 1 0 1)	(40 1 0 1)	(40 1 0 1)
*RMSEP*	0.677	0.573	0.689	0.619	0.610
					
**robustness (both)**					
Dec. Var.	(30 4 2 1)	(40 1 0 2)	(40 2 0 2)	(30 1 0 2)	(25 3 2 1)
*RMSEP*	0.289	0.360	0.427	0.457	0.391

a Dec. Var.: decision variables **u**^*T*^ = (*ws op od α*). *RMSEP*: root mean squared error prediction in the validation set.

### 5.3 Example from NIR Spectroscopy

Herein, we test our approach using another example from NIR spectroscopy taken from the literature.^42,45^ Both references include some type of wavelength selection, which is not considered in the present study. What is particularly interesting with this example is that the predictions for a standard PLS model built upon a typical accuracy objective fail to predict the process behavior for one particular data set, called test set 1 throughout this example, see Figure 7 in Muñoz and Torres.^45^

As a first step, we solve an optimization problem for accuracy using the complete data set as the training set and test set 1 as a validation set. The optimization resulted in the decision variables vector **u**^*T*^ = (20 1 0 1) and a value of ∼1.108 for *RMSEP*. As this PLS model is built explicitly on the prediction of test set 1, it is expected to yield the best prediction we could obtain by using a PLS regression model for this particular data set within the considered domain of decision variables.

In a next step, we randomly divide the data set into 70% training set and 30% test set and repeat again the optimization for different objectives. Note that now in all of these cases the optimization is agnostic with respect to test set 1, and we only use it as an independent test set, i.e., we check at the end of each optimization the obtained model and preprocessing process for the accuracy value in that particular test set.

For the robustness objective, we perform moment matching considering all available sources of variability both independently and combined. Those are paddle speed (PS), relative humidity (RH), instrument (Instr.), and mass rate (MR). Note that in all robustness cases a weight factor (β = 10^–4^) between the first and the second moment distances is considered since the order of magnitude for the second moment is significantly larger. Additional weights of 0.5 for PS and 25 for MR are also introduced for an even contribution from the different variability sources on the moment matching objective.

The results for all cases are summarized in Table 5. The results indicate that for the accuracy objective a total number of eight latent variables is chosen. The number of latent variables in the regression model is significantly overestimated compared to what we anticipate by looking at the results with test set 1 as the validation set (i.e., only one latent variable selected). When the robustness objective in all of the different variations is used, the optimization problem converges to a smaller number of latent variables, namely, one to three, which confirms the tendency of our robustness metric toward reducing model complexity. Regarding preprocessing, again small numbers for the window size of the SG filter are observed for accuracy and larger ones for the robustness. The optimizations with the robustness objective with respect to RH, Instr., and including all available sources of variability converge to a better solution in terms of predicting the test set than both the accuracy objective and the reference. Note that the decision variables from the reference are used here with the whole wave range (i.e., no wavelength selection step included), and thus, the reported values for the performance metrics are not expected to be identical with what would be obtained with the original model.

**Table 5:** Numerical Results for Optimization of Data Preprocessing and PLS Regression Using Different Objectives in the NIR Spectroscopy Example^a^

objective	Dec. Var.	*RMSEP*	*RMSEP_t_*
accuracy	(10 4 2 8)	0.414	1.917
robustness (PS)	(30 2 1 2)	2.168	3.824
robustness (RH)	(40 2 0 1)	3.681	1.155
robustness (Inst.)	(35 4 0 3)	1.963	1.624
robustness (MR)	(30 3 2 2)	2.031	3.710
robustness (all)	(40 3 1 3)	1.909	1.725
ref ^45^^b^	(11 2 1 3)	1.744	2.418

a Dec. Var.: decision variables **u**^*T*^ = (*ws op od α*). *RMSEP*: root mean squared error prediction in the validation set. *RMSEP*_*t*_: root mean squared error prediction in test set 1.

b The reference is to simulation with the empirical values of decision variables reported in Muñoz and Torres.^45^

By looking at the performance of the robustness objective for each individual source, we observe that just by considering RH or Inst. we obtain quite low values of *RMSEP* in the prediction of test set 1. Particularly, the results for robustness with respect to RH are similar to the results obtained when optimizing for the accuracy built on test set 1. It is therefore possible that a change in RH is responsible for the reference model not performing well in predicting test set 1 and could point to a direction of further investigation. In general, this observation demonstrates how the robustness metric applied across different known sources of variability can provide insight as to whether some of these sources can be responsible for a deterioration in model performance and help in identifying them. Overall, this shows high potential of our robustness metric not only as a performance indicator but additionally as a diagnostic tool.

**Figure 10:**
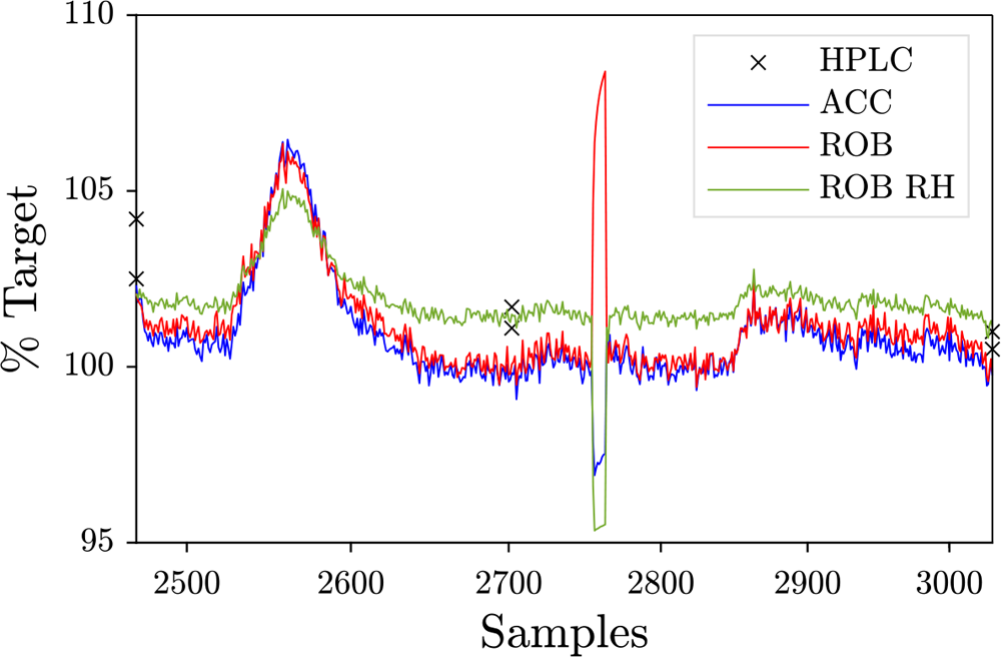
NIR spectroscopy example. Predictions by the different models on the independent test set 1, which is the data set not predicting well in the original reference by Muñoz and Torres.^45^ HPLC, HPLC measurements available. ACC, prediction based on the optimized for accuracy model. ROB, prediction based on the optimized for robustness with respect to all known sources of variability model. ROB RH, prediction based on the optimized for robustness with respect to relative humidity only model.

**Figure 11:**
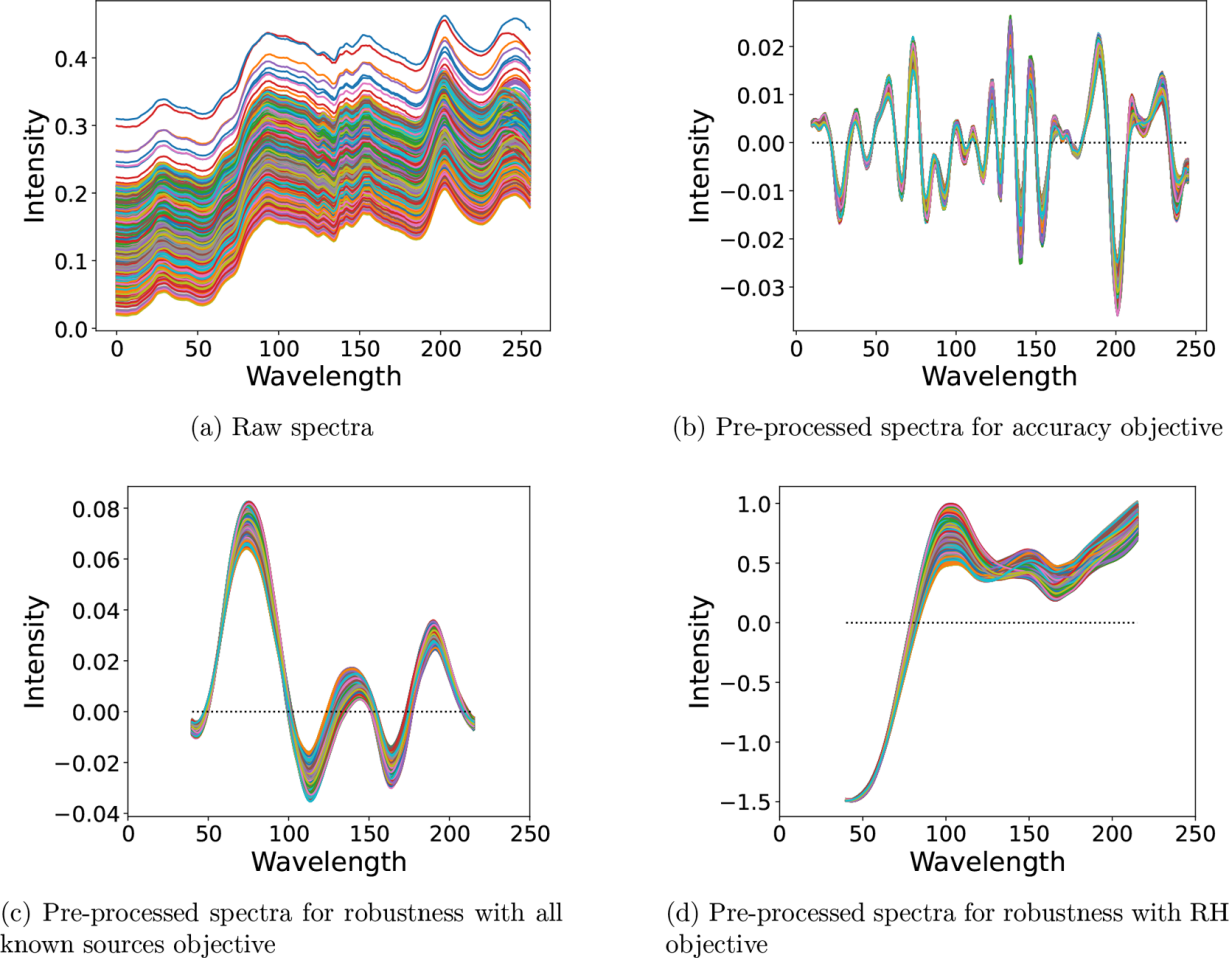
NIR spectroscopy example. (a) Raw and (b, c, and d) preprocessed (after SNV plus SG transform) spectra depicted for the training data set using optimal preprocessing from optimization with accuracy, robustness with all known sources of variation, and robustness with respect to relative humidity only, respectively

Figure 10 provides an illustration of the prediction of accuracy, robustness with all sources, and robustness with RH-only models in test set 1. Test set 1 consists of the HPLC points illustrated in Figure 10 that correspond to real experimental measurements at these points. The differences in the plot to Muñoz and Torres^45^ are due to the fact that in the reference the concentration of drug is calculated with respect to the formula without the nonresolved species (see Muñoz and Torres^45^ for more detail). We see that the robustness metric not only reduces model complexity but also predicts slightly better test set 1 than the accuracy one. As discussed, the model built for robustness on RH has the best performance for test set 1. Figure 11 shows the raw and preprocessed spectra for the corresponding cases. In this case, we believe that the accuracy model attempts to explain the API in every little peak, while robustness depending on what variation source it considers gives weight to diverse, more targeted spectral areas.

Complete enumeration for the above cases resulted to the same optimal solution as the one obtained from our optimizations, except from optimization with robustness with respect to Instr. For the last case, the difference in the objective value from compete enumeration and optimization was less than 1%, thus far below the expected predicted ability of the model.

As a next step, we repeat the optimizations with the examined objectives for different random splits of the data set into training and validation sets (keeping the 70–30% ratio constant). Note that split number one is the one used so far in the presented results, and unless stated otherwise, it is the one considered in the following.

The results are presented in Table 6. Overall, similar observations to the previous example (see section 5.2) can be made. In all cases examined, there seem to be some dependence of the obtained model to the random splits and consequently the considered data, which makes optimization a critical step.

**Table 6:** Numerical results for Optimization of Data Preprocessing and PLS Regression Using Different Random Splits for the Different Objectives in the NIR Spectroscopy Example^a^

objective	random 1	random 2	random 3	random 4	random 5
					
**accuracy**					
Dec. Var.	(10 4 2 8)	(10 4 2 8)	(15 4 0 8)	(10 4 2 8)	(10 4 0 8)
*RMSEP*	0.414	0.416	0.425	0.409	0.428
					
**robustness (PS)**					
Dec. Var.	(30 2 1 2)	(35 3 1 5)	(20 1 1 8)	(25 4 1 2)	(30 3 1 2)
*RMSEP*	2.168	0.984	0.470	1.925	2.086
					
**robustness (RH)**					
Dec. Var.	(40 2 0 1)	(40 2 1 1)	(40 2 0 1)	(40 4 1 5)	(40 2 0 1)
*RMSEP*	3.681	3.715	3.895	1.169	3.931
					
**robustness (Inst.)**					
Dec. Var.	(35 4 0 3)	(30 3 1 4)	(30 2 2 3)	(25 1 0 7)	(15 2 1 4)
*RMSEP*	1.963	1.402	1.889	0.587	1.480
					
r**obustness (MR)**					
Dec. Var.	(30 3 2 2)	(40 3 0 2)	(40 2 1 2)	(30 1 0 6)	(40 2 1 2)
*RMSEP*	2.031	2.734	2.586	0.660	2.771
					
r**obustness (all)**					
Dec. Var.	(40 3 1 3)	(35 2 2 5)	(40 3 2 3)	(30 3 2 5)	(40 4 1 5)
*RMSEP*	1.909	0.625	2.031	0.678	1.191

a Dec. Var.: decision variables **u**^*T*^ = (*ws op od α*). *RMSEP*: root mean squared error prediction in the validation set.

Finally, we compare the obtained models using some additional test sets available for this problem. More precisely, as we did previously, we first treat each test set as a validation set and perform optimization to find out what we should ideally expect in terms of optimal decision variables and *RMSEP* value. Then, we compare these results with the ones obtained from the models built for optimizing accuracy and robustness (with all available sources) objectives, respectively, while being agnostic to the existence of the corresponding test sets.

The outcomes of this comparison are illustrated in Table 7. For the majority of the test sets, only one latent variable would be required for the most accurate description (as resulted from the solution of the optimization problems). This means that for these cases a PLS model based solely on the accuracy objective would result to a much higher dimensionality of the latent space than actually needed. This could have a high effect on the overall predictability of the model and is possibly the reason the PLS model with three latent variables shown in Muñoz and Torres^45^ did not have a good performance for this case study. Yet, there are test sets, namely, 3, 7, where the “ideal” model would have a higher number of latent variables, and thus, for those the classic accuracy objective performs better. Overall, by using the robustness objective we anticipate a model with better prediction across multiple test sets, which is true for the majority of the test sets examined here.

**Table 7:** Comparison of PLS Model Performance Obtained from Optimization with Accuracy and Robustness Objectives on Different Validation Sets in the NIR Spectroscopy Example^a^

			*RMSEP_t_*	
test set	Dec. Var.	*RMSEP*	ACC (10 4 2 8)	ROB (40 3 1 3)
1	(20 1 0 1)	1.108	1.917	1.725
2	(25 3 1 1)	1.641	3.809	3.573
3	(15 1 1 8)	2.151	3.152	4.159
4	(40 2 1 1)	2.523	4.720	3.188
5	(10 2 2 1)	2.895	3.371	3.016
6	(10 2 2 1)	2.852	3.590	4.956
7	(15 4 2 8)	2.279	2.737	3.350

a Test set: test set. Dec. Var.: decision variables **u**^*T*^ = (*ws op od α*). *RMSEP*: root mean squared error prediction in the validation set. *RMSEP*_*t*_*ACC* (10 4 2 8): root mean squared error prediction evaluated with the accuracy model on the examined test set. *RMSEP*_*t*_*ROB* (40 3 1 3): root mean squared error prediction evaluated with the robustness model for all sources of variation on the examined test set.

## 6 Conclusion and Outlook

In this work, we investigate the automation of data preprocessing and model regression by incorporating them both simultaneously into an optimization step. The optimization problem yields optimal decision variables for data pretreatment and an optimal number of latent components for the underlying latent variable regression model. These optimal decisions are obtained based on either the accuracy or the robustness performance of the generated model. Model accuracy is evaluated on the difference between model predictions and observed values for the response, while a novel metric for quantifying robustness to known variability in the data is introduced. Our robustness definition utilizes the method of moments to minimize distances in the sample means and variances across different realizations of a known variability source.

Our method and performance metrics are tested with different examples from spectroscopy. The results show the dependency of the model building process to the available data, thus establishing optimization as a critical tool for decision making. As data preprocessing and model selection are performed sequentially in current state of the art approaches, our method can be used toward automation in building and updating chemometric models. In addition, the results demonstrate that models built solely on an accuracy objective usually have many components and tend to overfit the data, while models built on the robustness objective have lower complexity and are in general less amenable to overfitting. These characteristics of the robustness models are expected to reduce model maintenance efforts. However, this comes in some cases together with an inferior performance in terms of model prediction. Apart from the potential of the proposed robustness metric as a performance indicator, we also provide an example (see section 5.3) of how this metric can be additionally used as a diagnostic tool in cases where unusual model behavior occurs.

Further extensions are conceivable. Although this work accounts for a limited amount of preprocessing steps, it can be easily extended to include further options. The investigation of the sequence of the preprocessing steps as well as the times these are repeated could be also considered. Moreover, as the number of decision variables in the optimization problem increases, we anticipate an overall increase in the computational effort as well. Therefore, decomposition strategies or use of hierarchical programming is expected to play a key role in future investigations. Finally, although in this work we show only the extreme cases of building a model for maximizing either accuracy or robustness, in a practical scenario, good properties in both metrics are desired. In the future, robustness can be explicitly combined with the accuracy objective to generate models that are not overparametrized and have a high predictive ability. For this, deciding on the trade-off between the two objectives is identified as a challenging task.

## Supporting Information Available

Full list of abbreviations and symbols used in the manuscript; preprocessing and partial least-squares algorithm; robustness metric background theory (PDF)
