# Supplementary material for: An Optimization Approach Coupling Preprocessing with Model Regression for Enhanced Chemometrics
Source: Ind Eng Chem Res. 2023 Apr 5;62(15):6196–213. doi: 10.1021/acs.iecr.2c04583 (PMC10119938; doi:10.1021/acs.iecr.2c04583)
Supplement: Supplementary file 1 — ie2c04583_si_001.pdf [file ie2c04583_si_001.pdf]

Supplementary Information

An Optimization Approach Coupling Pre-processing with Model Regression for Enhanced Chemometrics

Chryssa Kappatou,† James Odgers,† Salvador García-Muñoz,‡ and Ruth Misener*,†

†*Computational Optimisation Group, Department of Computing, Imperial College London, London, SW7 2AZ, UK*

‡*Synthetic Molecule Design and Development, Lilly Research Laboratories, Eli Lilly & Company, Indianapolis, IN 46285, US*

Nomenclature

EMA European medicines agency

FDA United States food and drug administration

ICH international council for harmonisation of technical requirements for registration of pharmaceuticals for human use

$Instr.$ instrument

MR mass rate

PS paddle speed

RH relative humidity

API active pharmaceutical ingredient

PLS partial least squares

SG Savitzky-Golay

SNV standard normal variate

α number of latent variables

β weight factor between first and second moment distances

$\boldsymbol{\omega}$ pre-processing parameter vector

$\hat{\mathbf{X}}'^{*}$ model prediction of $\mathbf{X}'$; mean-centered

$\hat{\mathbf{X}}'$ model prediction of $\mathbf{X}'$; uncentered

$\hat{\mathbf{Y}}'^{*}$ model prediction of $\mathbf{Y}'$; uncentered

$\hat{\mathbf{Y}}'$ model prediction of $\mathbf{Y}'$; mean-centered

$\hat{\mathbf{Y}}_l$ model prediction of $\mathbf{Y}_l$; mean-centered

$\mathbf{E}$ random error in PLS input measurements

$\mathbf{e}$ column vector of all ones

$\mathbf{F}$ random error in PLS output measurements

$\mathbf{L}_1$ matrix with first moment distances

$\mathbf{L}_2$ matrix with second moment distances

$\mathbf{P}$ PLS loadings matrix for $\mathbf{X}$

$\mathbf{Q}$ PLS loadings matrix for $\mathbf{Y}$

$\mathbf{T}$ PLS scores matrix

$\mathbf{T}'$ PLS scores matrix for validation set

$\mathbf{t}_{k,PLS}$ input measurements in k^{th} iteration of NIPALS algorithm

$\mathbf{u}$ parameter vector; decision variables for the optimization problem

$\mathbf{W}^*$ modified PLS weight matrix

$\mathbf{W}$ PLS weights matrix

$\mathbf{X}$ input measurements after pre-processing; this is the input training set for the PLS model

$\mathbf{X}'$ input measurements after pre-processing; this is the input validation set for the PLS model

$\mathbf{X}'_m$ raw input measurements of the validation set

$\mathbf{X}_{k,PLS}$ input measurements in k^{th} iteration of NIPALS algorithm

$\mathbf{X}_l$ subcategory of input measurements that refers to the lth realization of a given variability source

$\mathbf{X}_m$ raw input measurements of the training set

$\mathbf{X}_{SG}$ input measurements of the training set after vector normalization and SG filter

$\mathbf{X}_{snv}$ input measurements of the training set after vector normalization

$\mathbf{Y}$ output measurements after mean-centering ; this is the output training set for the PLS model

$\mathbf{Y}'$ output measurements after mean-centering; this is the output validation set for the PLS model

$\mathbf{Y}'_m$ raw output measurements of the validation set

$\mathbf{Y}_{k,PLS}$ output measurements in k^{th} iteration of NIPALS algorithm

$\mathbf{Y}_l$ subcategory of output measurements that refers to the lth realization of a given variability source

$\mathbf{Y}_m$ raw output measurements of the training set

$\mathbf{z}$ vector with variability realizations

μ row-wise mean

σ row-wise variance

$\tilde{\mu}$ column-wise mean

$\tilde{\sigma}$ column-wise variance

d number of pairwise distances between each moment of a known source of variability

i row index

j column index

k iteration index for NIPALS algorithm

l index of instance realization for a known source of variability

m number of dependent variables

MM moment matching evaluated on the validation set; sum of $SL1 + SL2$; robustness objective

n number of independent variables after pre-processing

n_l number of possible realizations of a given variability source

o number of samples/observations in the validation set

od order of derivative

op order of polynomial

p number of frequencies measured by a spectrometer/ original number of independent variables

$RMSEP$ root mean squared error prediction evaluated on the validation set; accuracy objective

s number of samples/observations in the training set

s^2 sample variance

$SL1$ sum of first moment distances

$SL2$ sum of second moment distances

spe squared error prediction; evaluated on the validation set

ws window size

SA Pre-processing and Partial Least Squares algorithm

SA.1 Standard normal variate

Given a dataset with the raw input measurements of the training set, $\mathbf{X}_m \in \mathbb{R}^{s \times p}$, where s is the number samples or observations and p the number of measurement points, SNV applies the following row-wise transformation to the data

$$\mathbf{x}_{snv,i} = \frac{\mathbf{x}_{m,i} - \mu_{x,i}\mathbf{e}_1}{\sigma_{x,i}}, \quad \forall i \in [1, \ldots, s], \tag{S1}$$

where $\mathbf{e}_1 \in \mathbb{R}^{p \times 1}$ column vector of all ones, $\mu_{x,i}$ the row-wise mean and $\sigma_{x,i}$ the row-wise variance given by

$$\mu_{x,i} = \frac{1}{p}\sum_{j=1}^{p} x_{m,ij}, \quad \forall i \in [1, \ldots, s], \tag{S2}$$

$$\sigma_{x,i}^2 = \frac{1}{p-1}\sum_{j=1}^{p}(x_{m,ij} - \mu_{x,i})^2, \quad \forall i \in [1, \ldots, s]. \tag{S3}$$

SA.2 Mean-centering

Consider $\mathbf{X}_{SG} \in \mathbb{R}^{s \times n}$, obtained from $\mathbf{X}_m$ after SNV and SG transform, and the raw output training dataset, $\mathbf{Y}_m \in \mathbb{R}^{s \times m}$, where s is the number of samples or observations, n the number of independent variables ($n = p - 2ws$) and m the number of dependent variables. The input training dataset, $\mathbf{X} \in \mathbb{R}^{s \times n}$, is obtained after mean-centering similar to Equations (S1),(S2),(S3), but applied column-wise, with the column-wise variance set to one for all j

$$\mathbf{x}_j = \frac{\mathbf{x}_{SG,j} - \tilde{\mu}_{x,j}\mathbf{e}_1}{1}, \quad \forall j \in [1, \ldots, n], \tag{S4}$$

where $\mathbf{e}_1 \in \mathbb{R}^s$ column vector of all ones, $\tilde{\mu}_{x,j}$ the column-wise mean and $\tilde{\sigma}_{x,j}$ the column-wise variance given by

$$\tilde{\mu}_{x,j} = \frac{1}{s}\sum_{i=1}^{s} x_{SG,ij}, \quad \forall j \in [1, \ldots, n]. \tag{S5}$$

Equivalently, $\mathbf{Y}$ is obtained from the raw output measurements training set, $\mathbf{Y}_m \in \mathbb{R}^{s\times m}$, from

$$\mathbf{y}_j = \frac{\mathbf{y}_{m,j} - \tilde{\mu}_{y,j}\mathbf{e}_1}{1}, \quad \forall j \in [1,\dots,m], \tag{S6}$$

where

$$\tilde{\mu}_{y,j} = \frac{1}{s}\sum_{i=1}^{s} y_{m,ij}, \quad \forall j \in [1,\dots,m]. \tag{S7}$$

SA.3 NIPALS algorithm

The PLS model (shown in System (1)) can be obtained from utillizing NIPALS algorithm to solve the following parameter estimation problem:

$$\begin{aligned} \max_{\mathbf{w}_k} \quad & \mathbf{t}_{k,PLS}^T \mathbf{Y}_{k,PLS} \mathbf{Y}_{k,PLS}^T \mathbf{t}_{k,PLS} \\ \text{s.t.} \quad & \mathbf{t}_{k,PLS} = \mathbf{X}_{k,PLS}\mathbf{w}_k \\ & \mathbf{w}_k^T \mathbf{w}_k = 1, \end{aligned}$$

or equivalently by substituting $\mathbf{t}_{k,PLS}$ in the objective function

$$\begin{aligned} \max_{\mathbf{w}_k} \quad & \mathbf{w}_k^T \mathbf{X}_{k,PLS}^T \mathbf{Y}_{k,PLS} \mathbf{Y}_{k,PLS}^T \mathbf{X}_{k,PLS} \mathbf{w}_k \\ \text{s.t.} \quad & \mathbf{w}_k^T \mathbf{w}_k = 1, \end{aligned} \tag{S8}$$

for $k \in [1,\dots,\alpha]$, where $\mathbf{w}_k \in \mathbb{R}^{n\times 1}$, $\mathbf{t}_{k,PLS} \in \mathbb{R}^{s\times 1}$, $\mathbf{X}_{k,PLS} \in \mathbb{R}^{s\times n}$, $\mathbf{Y}_{k,PLS} \in \mathbb{R}^{s\times m}$. Definitions for the input, $\mathbf{X}_{k,PLS}$, and output, $\mathbf{Y}_{k,PLS}$, datasets for each iteration k of NIPALS algorithm are given by

$$\mathbf{X}_{k,PLS} = \begin{cases} \mathbf{X}, & \text{if } k = 1 \\ \mathbf{X}_{k-1,PLS} - \mathbf{t}_{k-1,PLS}\mathbf{p}_{k-1}^T, & \text{if } k \in (1,\alpha], \end{cases}$$

$$\mathbf{Y}_{k,PLS} = \begin{cases} \mathbf{Y}, & \text{if } k = 1 \\ \mathbf{Y}_{k-1,PLS} - \mathbf{t}_{k-1,PLS}\mathbf{q}_{k-1}^T, & \text{if } k \in (1, \alpha], \end{cases}$$

where the column vectors $\mathbf{p}_k \in \mathbb{R}^{n\times 1}$, $\mathbf{q}_k \in \mathbb{R}^{m\times 1}$ are evaluated by

$$\mathbf{p}_k = \frac{\mathbf{X}_{k,PLS}^T \mathbf{t}_{k,PLS}}{\mathbf{t}_{k,PLS}^T \mathbf{t}_{k,PLS}},$$

$$\mathbf{q}_k = \frac{\mathbf{Y}_{k,PLS}^T \mathbf{t}_{k,PLS}}{\mathbf{t}_{k,PLS}^T \mathbf{t}_{k,PLS}}.$$

At this point, it is crucial to notice that instead of solving Optimization Problem (S8) at each iteration, k, we can alternatively calculate the weight vector, $\mathbf{w}_k$, as the eigenvector associated with the largest eigenvalue of the $\mathbf{X}_{k,PLS}^T \mathbf{Y}_{k,PLS} \mathbf{Y}_{k,PLS}^T \mathbf{X}_{k,PLS}$ matrix using Singular Value Decomposition. However, as the size of $\mathbf{X}$ increases, the exact eigenvalue-eigenvector decomposition might become infeasible.[?]

After successful termination of NIPALS algorithm, we obtain

$$\mathbf{P} = [\mathbf{p}_1 \ldots \mathbf{p}_\alpha],$$

$$\mathbf{Q} = [\mathbf{q}_1 \ldots \mathbf{q}_\alpha],$$

$$\mathbf{W} = [\mathbf{w}_1 \ldots \mathbf{w}_\alpha],$$

with $\mathbf{W} \in \mathbb{R}^{n\times\alpha}$, the weights matrix. From these we can now evaluate

$$\mathbf{T} = \mathbf{X}\mathbf{W}^*,$$

where $\mathbf{W}^* \in \mathbb{R}^{n\times\alpha}$ is the modified weights matrix derived from

$$\mathbf{W}^* = \mathbf{W}(\mathbf{P}^T\mathbf{W})^{-1}.$$

By substitution of $\mathbf{T}$, $\mathbf{W}^*$ to the second equation of System (1), we get System (2).

SA.4 Predictions for validation set

Consider the $\mathbf{X}'_m \in \mathbb{R}^{o\times p}, \mathbf{Y}'_m \in \mathbb{R}^{o\times m}$, raw measurement input and output matrices, of the validation set. We first apply the pre-processing steps (see Section 2) to obtain $\mathbf{X}' \in \mathbb{R}^{o\times n}$ and $\mathbf{Y}' \in \mathbb{R}^{o\times m}$ the datasets describing the input and output measurements, respectively.

We can now obtain $\hat{\mathbf{X}}' \in \mathbb{R}^{o\times n}$ the reconstruction (prediction) of $\mathbf{X}'$ and $\hat{\mathbf{Y}}' \in \mathbb{R}^{o\times m}$ the prediction of $\mathbf{Y}'$ from

$$\mathbf{T}' = \mathbf{X}'\mathbf{W}^*,$$

$\mathbf{T}' \in \mathbb{R}^{o\times \alpha}$ and consequently

$$\hat{\mathbf{X}}' = \mathbf{T}'\mathbf{P}^T,$$

$$\hat{\mathbf{Y}}' = \mathbf{T}'\mathbf{Q}^T.$$

Note, however, that $\hat{\mathbf{X}}'$, $\hat{\mathbf{Y}}'$ are subject to the mean-centering process of the training dataset. Thus, to account for this we need to calculate $\hat{\mathbf{X}}'^* \in \mathbb{R}^{o\times n}$, $\hat{\mathbf{Y}}'^* \in \mathbb{R}^{o\times m}$ by solving for each $i \in [1, \ldots, o]$, $\hat{\mathbf{x}}'^*_i = \hat{\mathbf{x}}'_i + \tilde{\boldsymbol{\mu}}_x$, $\hat{\mathbf{y}}'_i + \tilde{\boldsymbol{\mu}}_y$, where $\tilde{\boldsymbol{\mu}}_x \in \mathbb{R}^{n\times 1}$ column vectors with each element $j \in [1, \ldots, n]$ obtained from Equation (S5) and $\tilde{\boldsymbol{\mu}}_y \in \mathbb{R}^{m\times 1}$ column vectors with each element $j \in [1, \ldots, m]$ obtained from Equation (S7).

SB Robustness metric background theory

This section theoretically justifies how the moment matching definition favors models with a small number of latent variables. We do this by showing that including components that make similar predictions for all realizations of a source of variability, will pull the moments of two selected variability groups apart with a probability of more than 50%. If the model outputs are drawn from the same distribution, it is therefore likely that our robustness model will be penalized for including an additional latent variable.

This proof only compare two groups along a single dimension. The same arguments apply for larger number of groups or output dimensions, as the results for these cases are just the addition of the one dimensional case with two groups.

Lemma 1. *Suppose a set of $A-1$ latent variables has produced a PLS model and we are considering to add an Ath latent variable. The output of the prediction from the Ath component is $\boldsymbol{y}_A$. For each of the samples, $\{y_{A,i}\}_{i=1}^{s}$ we assume $y_{A,i} \sim G_A$, where G_A is a random distribution with finite moments. We now assume that the samples $i \in \{1, \ldots, s\}$ are allocated into two sets $\mathcal{S}_l$, $l \in \{1, 2\}$, with $\mathcal{S}_1$ corresponding to all points with realization 1, and $\mathcal{S}_2$ corresponding to all points with realization 2 of the variability source. The nth moment of the predictions for an $A-1$ component model containing predictions from samples $\mathcal{S}_l$ is then $\mathfrak{M}^n_{A-1,l}$. The proof shows that $|\mathfrak{M}^n_{A,1} - \mathfrak{M}^n_{A,2}| > |\mathfrak{M}^n_{A-1,1} - \mathfrak{M}^n_{A-1,2}|$ with probability greater than 1/2, i.e., the absolute difference between the two n^{th} moments will probably increase with the introduction of the new latent variable.*

Without loss of generality, we label two different realizations of a source of variability, such that the nth moment of data with realization 1 ($\mathfrak{M}^n_{A-1,1}$) is greater than the nth moment of data with realization 2 ($\mathfrak{M}^n_{A-1,2}$).

The predictions form a PLS model come from adding the predictions from each of the individual predictions, so

$$y = \sum_{a=1}^{A} y_a, \tag{S9}$$

where y is the prediction from the complete model and y_a is the contribution from a single component. Similarly, we can break down the moment of the prediction from the set of predictions coming from samples coming from one realisation of a source of variability can be found by adding the contributions of the moments from each component:

$$\mathfrak{M}^n_{A,l} = \sum_{a=1}^{A} M^n_{a,l}, \tag{S10}$$

where $M^n_{A,l}$ is the contribution of the ath latent variable to the nth moment of data coming from source of variably l.

The nth sample moment of the contributions of the Ath latent variable to the predictions from group l, containing s_l samples, is given by

$$M^n_{A,l} = \frac{1}{s_l} \sum_{i \in \mathcal{S}_l} y^n_{A,i}. \tag{S11}$$

For a large number of samples, $M^n_{A,l}$ will be distributed according to $M^n_{A,l} \sim \mathcal{N}\left(\bar{g}_{A,n}, \frac{\sigma^2_{g_{A,n}}}{s_l}\right)$, where $\bar{g}_{A,n}$ is the expectation of y^n_A and $\sigma^2_{g_{A,n}}$ is the variance of y^n_A, by the Central Limit Theorem.

The nth moment of the prediction from the full PLS model is given by the sum of the nth moments of each component. Hence, after the new component is added to the robust model, the moments for the predictions of realization 1 ($\mathfrak{M}^n_{A,1}$) and realization 2 ($\mathfrak{M}^n_{A,2}$) are given by

$$\begin{aligned} \mathfrak{M}^n_{A,1} &= M^n_{A,1} + \mathfrak{M}^n_{A-1,1}, \\ \mathfrak{M}^n_{A,2} &= M^n_{A,2} + \mathfrak{M}^n_{A-1,2}. \end{aligned} \tag{S12}$$

There are four possibilities for whether the absolute difference between the moments of realization 1 and realization 2 grows as we add another latent variable. The first option is that the initial moment from realization 1 is initially higher than realization 2 ($\mathfrak{M}^n_{A-1,1} > \mathfrak{M}^n_{A-1,2}$) and the contribution of the Ath latent variable is greater for realization 1 than

realization 2 (*i.e.* $M^n_{A,1} - M^n_{A,2} > 0$). The second option is that the initial moment from realization 1 is initially higher than realization 2 (*i.e.* $\mathfrak{M}^n_{A-1,1} > \mathfrak{M}^n_{A-1,2}$), but after adding the contribution from the Ath moment the difference between realization 2 is greater than realization 1 with a value larger than the initial difference - a condition which is met if $M^n_{A,1} - M^n_{A,2} < -2(\mathfrak{M}^n_{A-1,1} - \mathfrak{M}^n_{A-1,2})$. The other two conditions are equivalent, but with realization 2 initially being greater than realization 1. This allows the probability of increasing the differences between the moments of realization 1 and 2 to be written as

$$
\begin{aligned}
P(|\mathfrak{M}^n_{A,1} - \mathfrak{M}^n_{A,2}| > &|\mathfrak{M}^n_{A-1,1} - \mathfrak{M}^n_{A-1,2}|) = \\
&P(\mathfrak{M}^n_{A-1,1} - \mathfrak{M}^n_{A-1,2} > 0)P(M^n_{A,1} - M^n_{A,2} > 0) \\
&+P(\mathfrak{M}^n_{A-1,1} - \mathfrak{M}^n_{A-1,2} > 0)P(M^n_{A,1} - M^n_{A,2} < -2(\mathfrak{M}^n_{A-1,1} - \mathfrak{M}^n_{A-1,2})) \\
&+P(\mathfrak{M}^n_{A-1,1} - \mathfrak{M}^n_{A-1,2} < 0)P(M^n_{A,1} - M^n_{A,2} > 2(\mathfrak{M}^n_{A-1,1} - \mathfrak{M}^n_{A-1,2}) \\
&+P(\mathfrak{M}^n_{A-1,1} - \mathfrak{M}^n_{A-1,2} < 0)P(M^n_{A,1} - M^n_{A,2} < 0).
\end{aligned}
$$

Due to our labeling such that the nth moments of sample 1 are greater than those of sample 2 at the beginning of the proof, the probability of $\mathfrak{M}^n_{a-1,1}$ being greater than $\mathfrak{M}^n_{a-1,2}$ is one, i.e., $P(|\mathfrak{M}^n_{A-1,1} - \mathfrak{M}^n_{A-1,2}| > 0) = 1$ and $P(|\mathfrak{M}^n_{A-1,1} - \mathfrak{M}^n_{A-1,2}| < 0) = 0$. This simplifies the above expression to

$$
\begin{aligned}
P(|\mathfrak{M}^n_{A,1} - \mathfrak{M}^n_{A,2}| > |\mathfrak{M}^n_{A-1,1} - \mathfrak{M}^n_{A-1,2}|) =& P(M^n_{A,1} - M^n_{A,2} > 0) \\
&+ P(M^n_{A,1} - M^n_{A,2} < -2(\mathfrak{M}^n_{A-1,1} - \mathfrak{M}^n_{A-1,2})).
\end{aligned}
$$

We can then use the fact that $M^n_{A,1} - M^n_{A,2} \sim \mathcal{N}\left(0, \sigma^2_{g_{A,n}}\left(\frac{1}{s_1} + \frac{1}{s_2}\right)\right)$, which has an equal chance of being greater than or less than zero, to make the substitution

$$
\begin{aligned}
&= \frac{1}{2} + P(M^n_{A,1} - M^n_{A,2} < -2(\mathfrak{M}^n_{A-1,1} - \mathfrak{M}^n_{A-1,2})) \\
&> \frac{1}{2}. \square
\end{aligned}
$$
